# MRSA‐Triggered Disassembly of an Immune‐Recruiting NIR Probe for Bacterial Imaging and Clearance

**DOI:** 10.1002/advs.78522

**Published:** 2026-10-31

**Authors:** Jiacheng Du, Yongyi Chen, Ling Lei, Yunzhe Tao, Yu Peng, Qingyang Zhang, Qinghua Wang, Hai‐Yu Hu

**Affiliations:** ^1^ State Key Laboratory of Bioactive Substances and Function of Natural Medicine Institute of Materia Medica Chinese Academy of Medical Sciences and Peking Union Medical College Beijing China

**Keywords:** disassembly‐activated NIR fluorescent probe, methicillin‐resistant *Staphylococcus aureus*, theranostics, immune recruitment, ferroptosis‐like bacterial death

## Abstract

Methicillin‐resistant *Staphylococcus aureus* (MRSA) infections remain a major clinical challenge due to increasing antimicrobial resistance and limited strategies for precise bacterial recognition and clearance. Here, we report an immune‐recruiting supramolecular amphiphile, **Dman‐C11**, that undergoes MRSA‐triggered structural reorganization for activatable bacterial theranostics. **Dman‐C11** consists of a hemicyanine near‐infrared (NIR) fluorophore, a D‐mannosamine immune‐recruiting motif, and a hydrophobic C11 chain that promotes bacterial membrane association. The amphiphilic architecture drives spontaneous nanoparticle formation with suppressed fluorescence through aggregation‐caused quenching (ACQ). Upon interaction with the MRSA membrane, nanoparticle disassembly redistributes the probe within the lipid bilayer, producing a 20‐fold fluorescence turn‐on response while simultaneously restoring efficient singlet oxygen generation under 655 nm irradiation. The activated photodynamic process induces ferroptosis‐like bacterial death characterized by extensive lipid peroxidation and oxidative membrane damage. Meanwhile, the D‐mannosamine motif promotes immune‐cell recruitment toward bacterial pathogens, thereby facilitating host‐assisted bacterial clearance. In murine wound infection and bacterial keratitis models, **Dman‐C11** achieves approximately 20‐fold reductions in bacterial burden without detectable systemic toxicity. This membrane‐triggered supramolecular disassembly strategy establishes a single‐molecule theranostic approach that links bacterial recognition, activatable imaging, photodynamic antibacterial activity, and immune‐assisted clearance for precise treatment of MRSA infections.

## Graphical abstract

A bacteria–immune cell‐bridging supramolecular amphiphile, **Dman‐C11**, undergoes MRSA membrane‐triggered disassembly to activate NIR fluorescence and photodynamic killing, inducing ferroptosis‐like bacterial death and immune‐assisted clearance in wound infection and keratitis models.

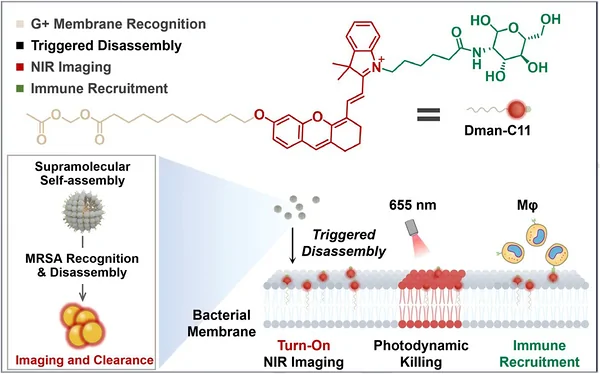

## 1 Introduction

The rapid emergence of antimicrobial resistance has become a major global health challenge [1, 2], with methicillin‐resistant *Staphylococcus aureus* (MRSA) representing one of the most prevalent causes of persistent skin, soft tissue, and ocular infections [3, 4]. The limited effectiveness of conventional antibiotics highlights the need for therapeutic approaches that enable precise bacterial recognition while providing localized antimicrobial activity [5, 6, 7]. Molecular theranostic systems that combine activatable imaging with antibacterial intervention offer an attractive strategy for improving treatment specificity and reducing off‐target toxicity [8, 9]. NIR fluorescence imaging enables real‐time, in situ visualization of pathogens with deep tissue penetration, but conventional probes often suffer from low signal‐to‐noise ratios, making activatable probes preferable for precise detection [10, 11, 12, 13]. Antimicrobial photodynamic therapy (aPDT) kills bacteria locally through photoinduced reactive oxygen species with minimal risk of resistance [14, 15]. This approach has shown great promise in the treatment of localized infections, such as skin wounds, corneal infections, and oral lesions. Ferroptosis, a regulated form of cell death driven by lipid peroxidation and ROS accumulation, has been widely studied in cancer therapy under photodynamic conditions, but its role in bacterial killing remains largely unexplored. Mimicking ferroptosis‐like mechanisms could improve the effectiveness of photodynamic antibacterial strategies [16, 17, 18].

The cytoplasmic membrane of *S. aureus*, composed primarily of anionic phospholipids such as phosphatidylglycerol and cardiolipin, constitutes a key interface for molecular recognition and antibacterial intervention. Lipid‐mimetic motifs can insert into bacterial membranes, perturb lipid packing, and increase membrane permeability [19]. Building on this, conjugation of lipid chains to probes or antibiotics enhances membrane affinity and drives selective accumulation at bacterial sites [20, 21]. In aggregation‐caused quenching (ACQ) systems, probes are initially self‐assembled and non‐emissive; upon membrane insertion, they disassemble and redistribute on the membrane, restoring fluorescence and improving the signal‐to‐background ratio [22, 23, 24]. While such membrane‐targeting strategies are well studied in cancer systems, their use in bacterial imaging and therapy, particularly against MRSA, remains limited [25, 26, 27, 28].

Besides direct bacterial killing, activation of host innate immunity provides an additional mechanism for eliminating residual pathogens and accelerating infection resolution [29, 30, 31, 32]. Mannosylated scaffolds and poly‐D‐mannose derivatives have been shown to recruit immune cells and facilitate immune‐mediated bacterial clearance [33, 34, 35]. Incorporating these mannosylated motifs into therapeutic agents offers a strategy to engage host immunity against MRSA, yet this approach remains largely unexplored. Combating multidrug‐resistant infections further requires systems that enable precise pathogen detection and localized intervention. While multifunctional nanomaterials have been widely studied, their structural heterogeneity and limited reproducibility constrain translational potential [36, 37]. Single‐molecule, metal‐free organic probes, in contrast, offer defined structures, tunable properties, predictable biological interactions, and improved biocompatibility, tissue penetration, and translational applicability.

Here, we report **Dman‐C11**, an amphiphilic NIR fluorescent probe comprising a hemicyanine dye conjugated to a D‐mannosamine segment and a hydrophobic alkyl chain. **Dman‐C11** self‐assembles into nanoparticles exhibiting ACQ, which selectively disassembles upon interaction with MRSA membranes, restoring NIR fluorescence and enabling efficient ROS generation under 655 nm irradiation (Scheme 1). The resulting photodynamic effect induces ferroptosis‐like bacterial death, while the D‐mannosamine segment simultaneously promotes immune cell recruitment, enhancing bacterial clearance in both wound and corneal infection models. By coupling membrane‐triggered disassembly with fluorescence activation, photodynamic killing, and immune recruitment, **Dman‐C11** exemplifies a single‐molecule, immune‐engaging theranostic platform for combating multidrug‐resistant bacterial infections.

**SCHEME 1:**
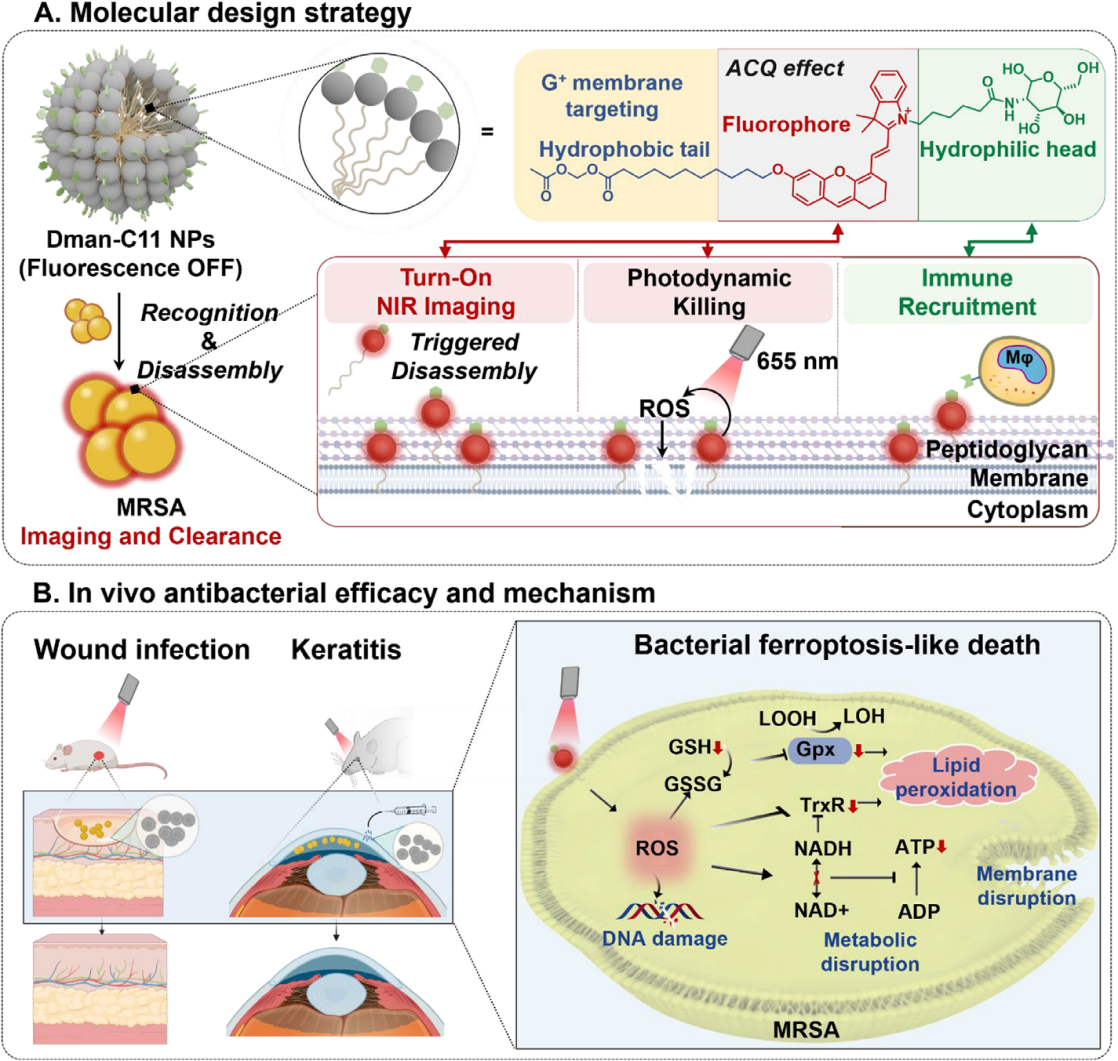
Schematic illustration of **Dman‐C11 NPs**. (A) Molecular design strategy of ACQ‐active **Dman‐C11** probe for bacteria‐selective imaging and immune‐assisted photodynamic antibacterial activity. (B) In vivo demonstration of bacteria‐selective antibacterial activity of **Dman‐C11 NPs** in wound infection and bacterial keratitis models, and the molecular mechanism underlying the antibacterial activity.

## 2 Results and Discussion

### 2.1 Design, Synthesis, and Self‐Assembly‐Regulated Photophysical Properties of **Dman‐C11**

To develop a mannose‐functionalized hemicyanine probe with bacterial membrane affinity, immune recruitment capability, NIR optical properties, and programmable self‐assembly, we designed and synthesized **Dman‐C11** (Figure 1A). The probe is based on a cationic donor–acceptor (D–A) hemicyanine scaffold, enabling NIR absorption and efficient singlet oxygen (^1^O_2_) generation [38]. A D‐mannosamine moiety was introduced for immune recognition, while a hydrophobic alkyl chain was incorporated to enhance bacterial association. The synthetic route is provided in Scheme S1.

**FIGURE 1:**
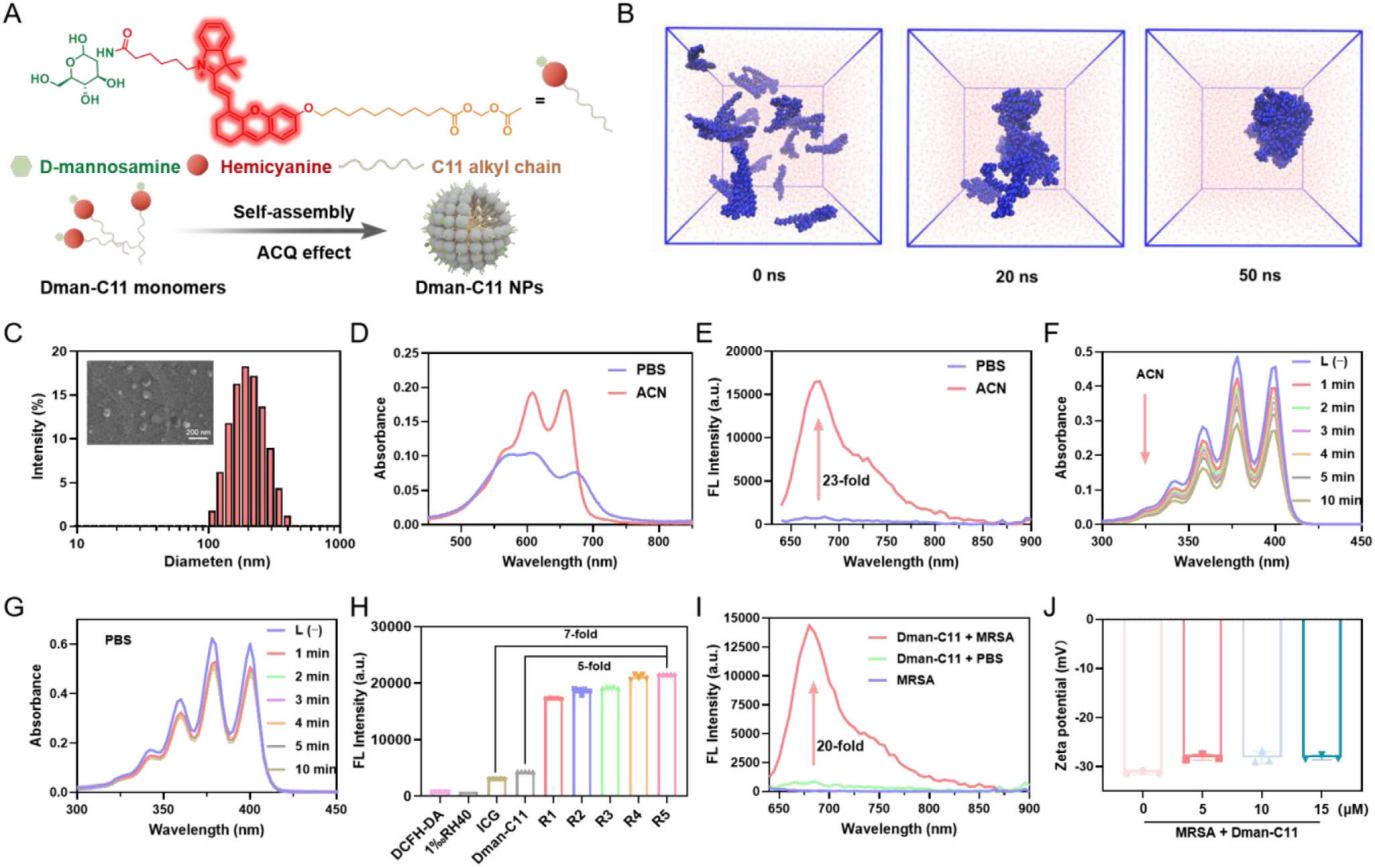
Characterization of **Dman‐C11**. (A) Schematic illustration of **Dman‐C11**. (B) MD simulations of **Dman‐C11**. (C) Particle size distributions of **Dman‐C11**; inset, SEM image of **Dman‐C11**. (D) Absorption and (E) Fluorescence spectra of **Dman‐C11** in PBS and ACN. (F,G) ABDA degradation in the presence of **Dman‐C11** in ACN (F) and (G) PBS under irradiation. (H) Fluorescence spectra of DCF in PBS, R1‐R5: 0.1‰, 0.3‰, 0.5‰, 0.8‰, and 1.0‰ RH40 + **Dman‐C11**; ICG: positive control; 0.1% RH40: negative control. Data are presented as mean ± s.d. (*n* = 3). (I) Fluorescence spectra of MRSA, **Dman‐C11**, and **Dman‐C11** + MRSA. (J) Zeta potential of MRSA incubated with **Dman‐C11** at different concentrations, (*n* = 3).

Molecular dynamics (MD) simulations revealed spontaneous self‐assembly of **Dman‐C11** into nano‐aggregates in aqueous buffer (Figure 1B) [39]. Consistently, scanning electron microscopy (SEM) and dynamic light scattering (DLS) confirmed the formation of well‐defined nanoparticles with an average hydrodynamic diameter of 188 nm and a low polydispersity index (PDI = 0.172, Figure 1C). **Dman‐C11** exhibited a broad absorption band from 500 to 700 nm and an emission maximum at 680 nm (Figure 1D). Its fluorescence was nearly quenched in PBS due to ACQ, while a ∼23‐fold enhancement was observed in acetonitrile (ACN) where the probe is molecularly dispersed (Figure 1E). Upon incubation with MRSA, **Dman‐C11** nanoparticles (**Dman‐C11 NPs**) showed ∼20‐fold fluorescence enhancement (Figure 1I), with zeta potential measurements indicating minimal changes in bacterial surface charge and suggesting hydrophobic interactions as the primary driving force for probe‐bacteria association (Figure 1J, Figure S1). Upon incubation with MRSA, **Dman‐C11 NPs** exhibited a 20‐fold fluorescence enhancement (Figure 1I). Only minor changes in zeta potential were observed after incubation, suggesting that electrostatic interactions are not the primary driving force for probe association with MRSA (Figures 1J and Figure S1). To further examine the role of hydrophobic interactions, **Dman‐C11 NPs** were treated with 1,4‐dioxane (10%–95%) or Polysorbate 20 (Tween 20; 0.5‰–5‰). Both treatments induced dose‐dependent fluorescence recovery, consistent with disruption of hydrophobic interactions and nanoparticle disassembly (Figure S2). In addition, the fluorescence signal remained stable during 20 min of incubation with MRSA, and the emission spectra in PBS and ACN showed no appreciable changes after laser irradiation (Figure S3). These findings indicate that the fluorescence enhancement arises from MRSA‐triggered nanoparticle disassembly and fluorescence dequenching. This aggregation‐dependent behavior also regulated photoinduced ROS generation. Using ABDA as a singlet oxygen (^1^O_2_) indicator under 655 nm irradiation, negligible ABDA consumption was observed in the aggregated state, whereas efficient degradation occurred in ACN (Figure 1F,G). ROS generation was further evaluated using the DCFH‐DA assay, monitored by oxidation of DCFH to fluorescent DCF. Indocyanine green (ICG) was used as a positive control, RH40 (0.1%–1‰) was used to provide a micellar environment that promotes disassembly of **Dman‐C11 NPs**, consistent with the disassembly expected upon association of **Dman‐C11** with the bacterial membrane. Under 655 nm irradiation, **Dman‐C11** in 1‰ RH40 exhibited ∼five fold higher DCF fluorescence than in PBS and ∼seven fold higher than ICG (Figure 1H), demonstrating enhanced ROS generation upon disassembly [40]. These results indicate that **Dman‐C11** is quenched and photodynamically inactive in the aggregated state, but becomes activated upon MRSA‐mediated disassembly, enabling site‐specific fluorescence and ROS generation for precise bacterial detection.

### 2.2 Highly Selective Turn‐On Imaging of Gram‐Positive Bacteria In Vitro and In Vivo

Confocal laser scanning microscopy (CLSM) revealed strong fluorescence activation of **Dman‐C11 NPs** in Gram‐positive bacteria, while negligible signals were observed in mammalian cells (HepG2 and HK‐2) and Gram‐negative bacteria (Figure 2A, B). High‐contrast images showed membrane‐localized accumulation, consistent with disassembly‐induced fluorescence activation (Figure 2C,D). These results demonstrate the high selectivity of **Dman‐C11 NPs** for Gram‐positive bacteria.

**FIGURE 2:**
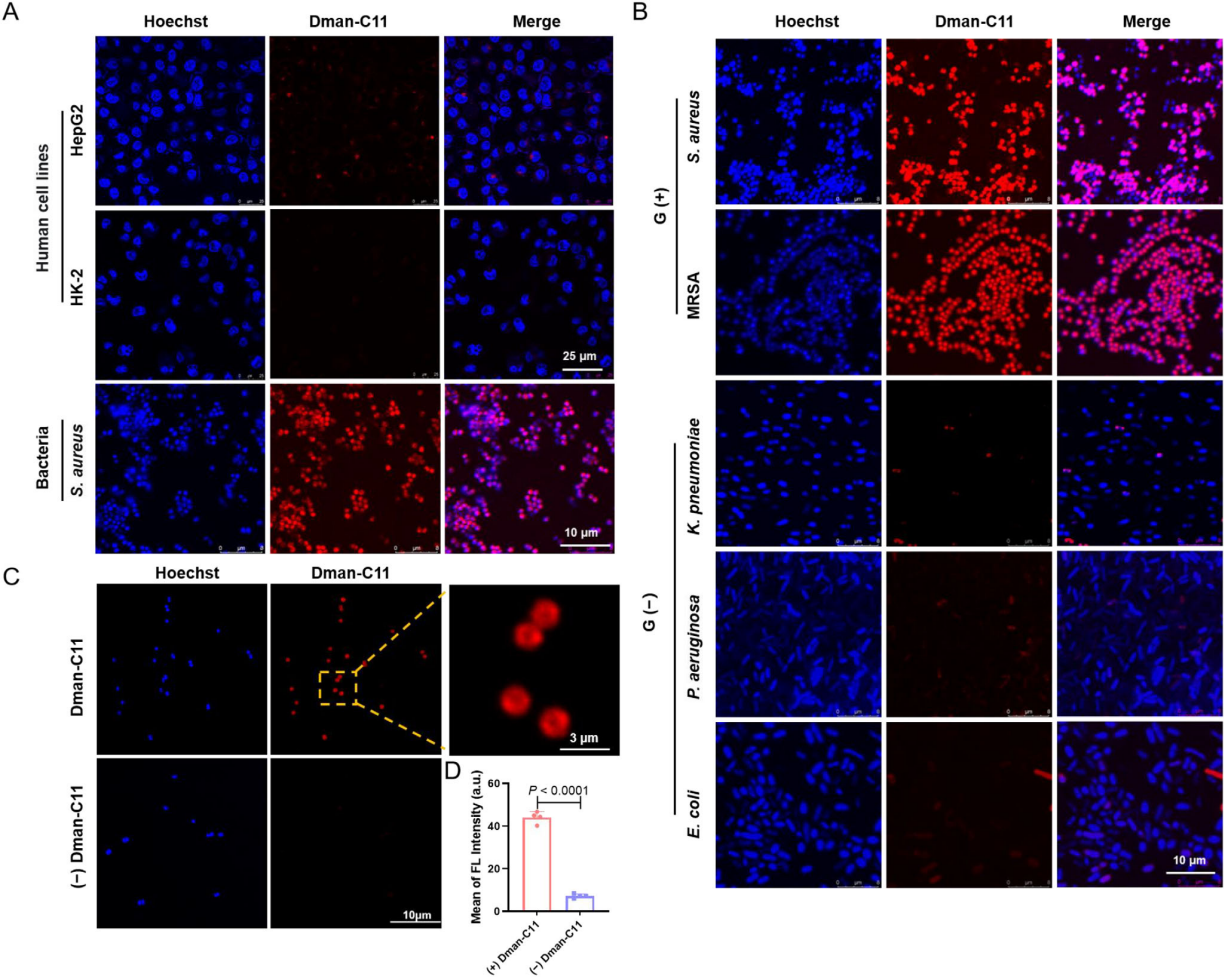
Confocal fluorescence imaging of **Dman‐C11 NPs** in cells and bacteria. (A) *S. aureus* (*Staphylococcus aureus*) and mammalian cells (HK‐2, HepG2). (B) Representative Gram‐positive and Gram‐negative bacteria. (C) Membrane‐localized fluorescence in bacteria. (D) Quantification of fluorescence intensity with and without **Dman‐C11 NPs**. Data are presented as mean ± s.d. (*n* = 4). Statistical significance was determined using an unpaired, two‐sided Student's *t*‐test (*P* < 0.0001).

Hemolysis and cytotoxicity assays showed minimal red blood cell (RBC) lysis up to 25 µM and > 90% cell viability across normal and cancer cell lines, indicating good biocompatibility (Figures S4 and S5). In a murine model with MRSA infection (right thigh) and LPS‐induced inflammation (left), **Dman‐C11 NPs** were intravenously administered 18 h post‐infection. Rapid accumulation at the infection site led to fluorescence activation within 1 h, peaking at 3 h and declining by 5 h, with no detectable signal in inflamed tissue. (Figure 3A–C and Figure S6). Ex vivo imaging revealed strong fluorescence at the infection site and liver, consistent with hepatic distribution (Figure S7a).

**FIGURE 3:**
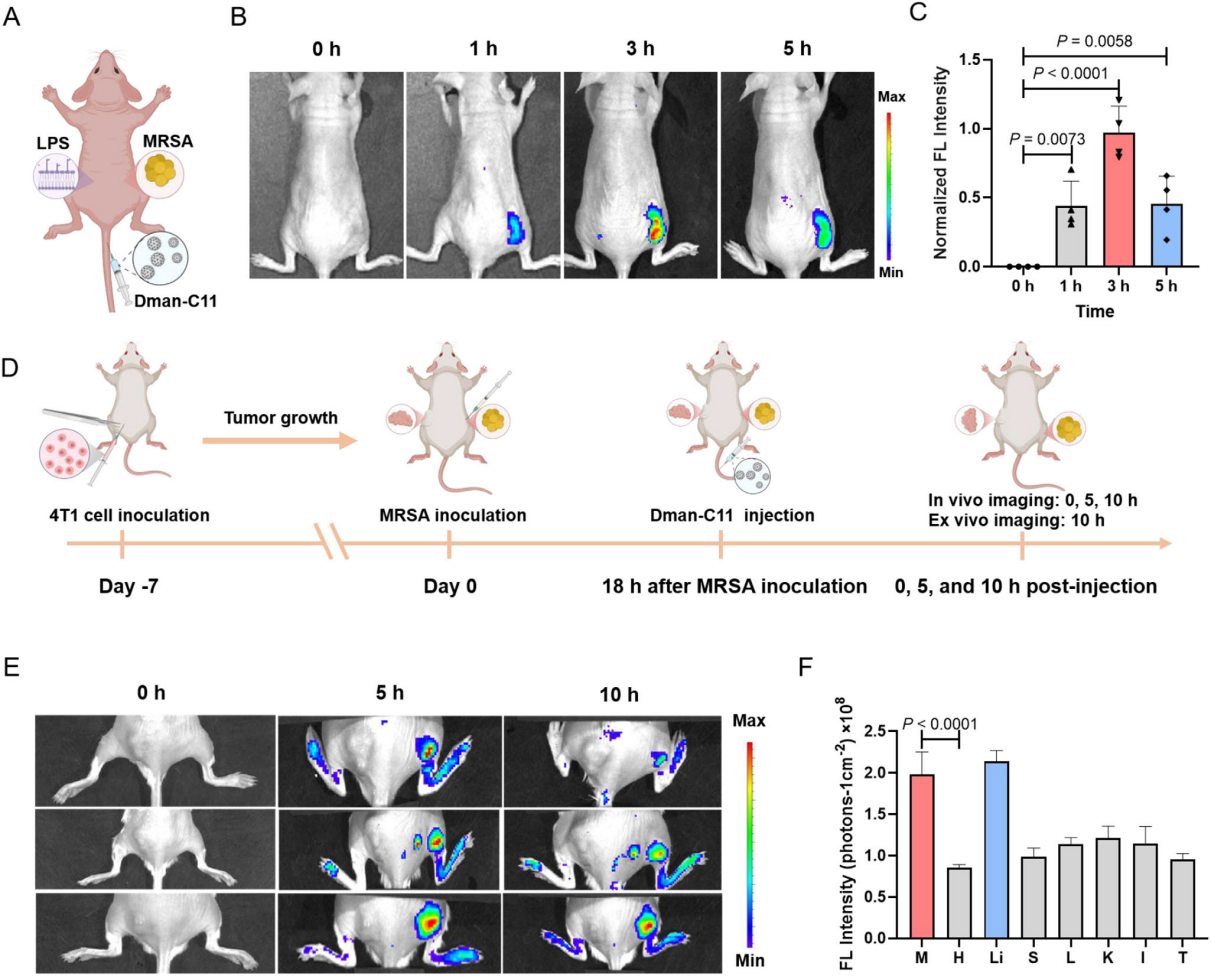
In vivo bacterial targeting and fluorescence imaging of **Dman‐C11 NPs**. (A–C) MRSA infection and sterile inflammation model: schematic (A), time‐dependent in vivo fluorescence images following i.v. injection of **Dman‐C11 NPs** (B), and quantification of fluorescence intensity (C). Data are presented as mean ± s.d. (*n* = 3 mice per group). (D–F) 4T1 tumor and MRSA coexistence model: schematic (D), time‐dependent NIR fluorescence images following i.v. injection of **Dman‐C11 NPs** (E), and ex vivo quantification of fluorescence intensity in major organs and tissues at 10 h post‐injection (F). M, MRSA‐infected site; H, heart; Li, liver; S, stomach; L, lung; K, kidney; I, intestine; T, tumor. Data are mean ± s.d. (*n* = 3 mice per group). One‐way ANOVA with Tukey's multiple‐comparison test.

In a tumor‐infection coexistence model (4T1 vs MRSA), tumors were allowed to grow for 7 days until reaching approximately 100 mm^3^, followed by MRSA infection. After 18 h of infection, **Dman‐C11 NPs** were administered via intravenous injection (100 µL, 25 µM). At 5 h post‐injection, the probe selectively accumulated at the infected site, with minimal signal observed in tumor tissue (Figure 3E). Consistently, ex vivo imaging at 10 h post‐injection showed dominant fluorescence in infected tissue and liver (Figure 3F, Figure S7b). These results demonstrate the high sensitivity and specificity of **Dman‐C11 NPs** for in vivo bacterial imaging, enabling discrimination of MRSA infection from sterile inflammation and tumors.

### 2.3 Immune Recruitment and PDT Dual‐Function Anti‐MRSA Activity

To evaluate immune recognition and bacterial clearance induced by **Dman‐C11**, both in vitro and in vivo studies were performed. In vitro assays were conducted using RAW264.7 macrophages and enhanced GFP (eGFP)‐expressing *S. aureus*. Bacteria were divided into three groups: untreated bacteria co‐incubated with untreated macrophages (control), bacteria labeled with **Dman‐C11** for 1 min followed by removal of unbound probe and incubation with untreated macrophages, and **Dman‐C11**‐labeled bacteria subjected to CD206 blockade by incubation with D‐mannosamine (Dman)‐pretreated RAW264.7 macrophages. CD206 expression on RAW264.7 macrophages was confirmed and increased following MRSA stimulation (Figure S8). After washing away non‐adherent bacteria, macrophage‐bacteria interactions were quantified by flow cytometry and visualized by confocal laser scanning microscopy (Figure 4A–C). Compared with the control group, **Dman‐C11**‐labeled bacteria exhibited significantly enhanced macrophage association and uptake. This effect was markedly reduced in the D‐man‐blocked group, confirming CD206‐dependent carbohydrate recognition. Consistently, CFU assays showed enhanced bacterial clearance in the **Dman‐C11** group, corresponding to an obvious increase in bacterial elimination compared with both the blocking and control groups (Figure 4D). Extending to in vivo validation, immunofluorescence analysis of infected tissues revealed markedly stronger CD206 and CD86 signals in the **Dman‐C11**‐treated MRSA group, indicating enhanced macrophage recruitment and activation at the infection site compared with untreated bacteria (Figure 4E).

**FIGURE 4:**
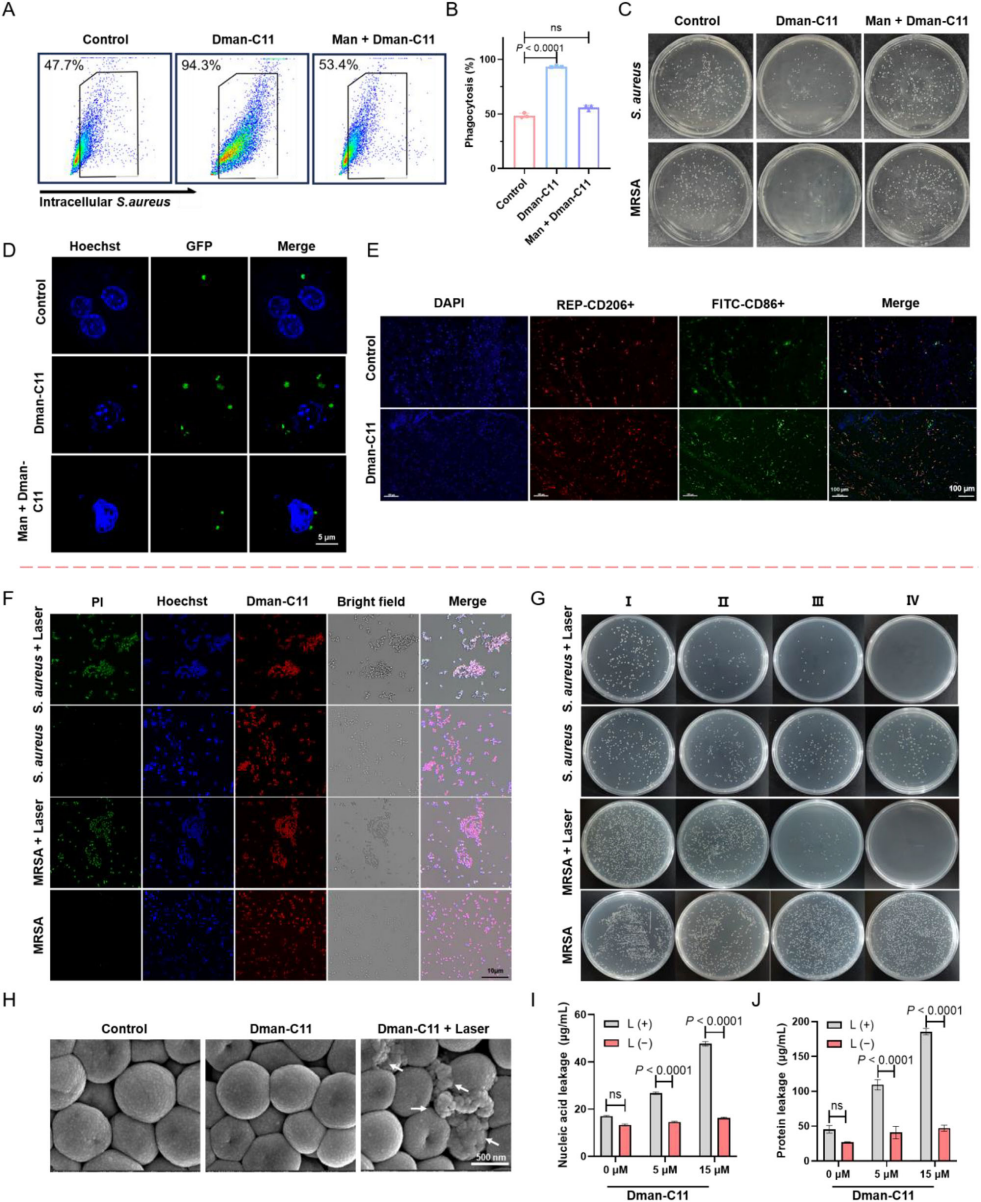
Immune recruitment and photodynamic antibacterial activity of **Dman‐C11 NPs**. (A,B) Flow cytometry profiles and quantification of macrophage phagocytosis of GFP‐expressing (*Staphylococcus aureus*) *S. aureus* under different treatment conditions. (C) Agar plate images showing bacterial survival of *S. aureus* and MRSA after co‐incubation with RAW264.7 macrophages. (D) Confocal images of macrophage‐mediated phagocytosis. Blue: nuclei; green: GFP signal; scale bars, 5 µm. (E) Immunofluorescence analysis of CD206 and CD86 in MRSA‐infected mouse tissues at 24 h post‐treatment. Blue: nuclei; red: CD206; green: CD86; scale bars, 100 µm. (F,G) Confocal images and CFU assays of *S. aureus* and MRSA treated with **Dman‐C11 NPs** with or without NIR irradiation I–IV: 0, 0.2, 1, 5 µM **Dman‐C11 NPs**, respectively (H–J) SEM images, protein leakage, and nucleic acid leakage of MRSA after **Dman‐C11 NPs** treatment with NIR irradiation. Data are mean ± s.d. (*n* = 3). One‐way ANOVA with Tukey's test (B); two‐way ANOVA with Bonferroni's test (I,J).

Under NIR irradiation (655 nm, 200 mW cm^−2^), **Dman‐C11** (1 µm) showed potent photodynamic antibacterial activity, as demonstrated by PI staining and CFU assays, with an approximately 7.5‐fold reduction in bacterial viability (Figure 4 F,G). **Dman‐C11** also induced fluorescence activation in additional *S. aureus* strains, including ATCC 25923, RN 6390, ATCC 29213, and a laboratory‐induced VRSA derivative of ATCC 29213, and showed photodynamic antibacterial activity against these strains under the same irradiation conditions (Figure S9). Enhanced DiBAC_4_(3) fluorescence indicated membrane depolarization, reflecting dissipation of the transmembrane potential. This effect was accompanied by pronounced leakage of intracellular nucleic acids and proteins and severe membrane disruption observed by SEM (Figure 4H–J and Figure S11), indicating photodynamically induced membrane damage and loss of membrane integrity. These results show that **Dman‐C11 NPs** couple pathogen targeting with CD206‐mediated immune recruitment and photodynamic bacterial killing, leading to enhanced bacterial clearance.

### 2.4 Dman‐C11‐Mediated Photodynamic Ferroptosis‐Like Process in MRSA

To elucidate the antibacterial mechanism of **Dman‐C11 NPs**, RNA sequencing (RNA‐seq) was performed on MRSA treated with **Dman‐C11 NPs** with or without NIR irradiation. Volcano plot analysis revealed substantial transcriptional remodeling upon NIR activation (Figure 5A), with 412 differentially expressed genes (DEGs) identified, including 248 upregulated and 164 downregulated genes. Gene Ontology (GO) and Kyoto Encyclopedia of Genes and Genomes (KEGG) pathway analyses indicated enrichment in metabolic pathways, secondary metabolite biosynthesis, membrane‐associated components, and metal ion binding (Figure 5B,C). Genes involved in glycolysis and pyruvate metabolism were significantly downregulated (Figure S12), suggesting impaired hypoxia adaptation. Key tricarboxylic acid (TCA) cycle genes (*sucC*, *oforA*, *oforB*, and *gltA*) were downregulated, indicating reduced ATP production, whereas *sdhA‐C* were upregulated, consistent with ROS accumulation (Figure 5D). Oxidative phosphorylation genes were broadly dysregulated (Figure 5E), and F‐type ATPase genes (*atpA‐H*) were upregulated, indicative of compensatory responses to energy imbalance. Lipid metabolism genes (*lip*, *gpx*, *fadD*) were also significantly altered (Figure 5F), suggesting disrupted lipid homeostasis.

**FIGURE 5:**
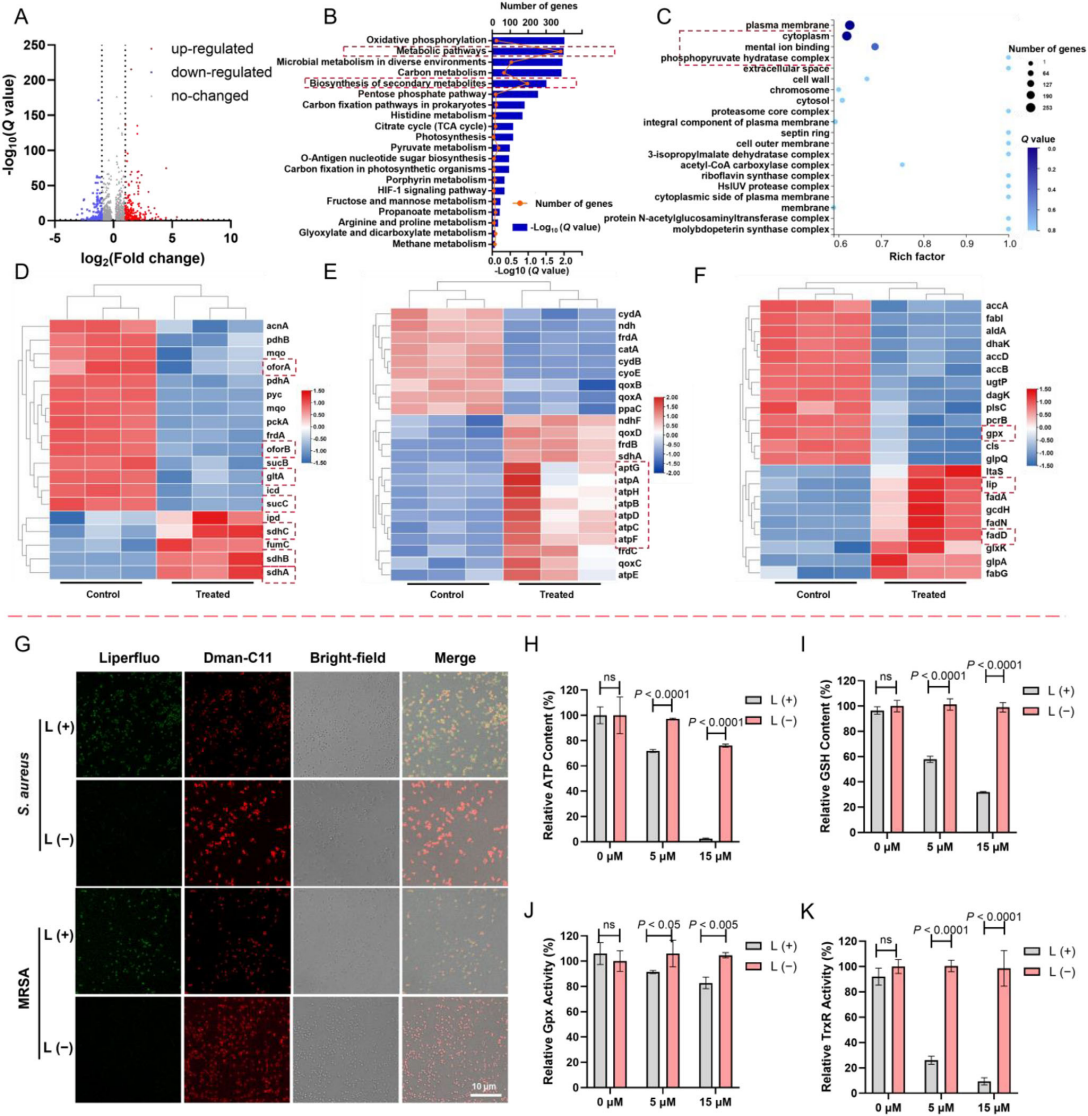
Transcriptomic profiling and PDT‐triggered metabolic and redox dysfunction in MRSA induced by **Dman‐C11 NPs**. (A) Volcano plot of DEGs between control and **Dman‐C11** NP‐treated MRSA. Red and blue dots indicate significantly upregulated and downregulated genes, respectively (|log_2_FC| > 1 and *Q* < 0.05; *n* = 3 biological replicates per group). (B,C) KEGG pathway (B) and GO term (C) enrichment analyses. In (B), blue bars indicate the number of candidate genes and orange dots indicate −log_10_(*Q* value). In (C), dot size indicates gene number and color indicates *Q* value. Enrichment analyses used Benjamini–Hochberg correction; *Q* < 0.05 was considered significant. (D–F) Heatmaps of DEGs related to the TCA cycle (D), oxidative phosphorylation (E), and lipid metabolism (F). Colors indicate row‐scaled Z‐scores of normalized expressions (*n* = 3 biological replicates per group). (G) CLSM images of Liperfluo‐stained (*Staphylococcus aureus*) *S. aureus* and MRSA treated with **Dman‐C11 NPs** with or without NIR irradiation. Scale bars, 10 µm. (H–K) Intracellular ATP (H), GSH content (I), Gpx activity (J), and TrxR activity (K) in MRSA treated with different concentrations of **Dman‐C11** with (L+) or without (L−) 655 nm irradiation. Data are normalized to the 0 µm L(−) group. Data are mean ± s.d. (*n* = 3). Two‐way ANOVA with Bonferroni's multiple‐comparison test.

To validate the transcriptomic findings, lipid peroxidation was first examined using Liperfluo and was markedly increased in bacteria treated with **Dman‐C11 NPs** under NIR irradiation (Figure 5G), indicating pronounced oxidative membrane damage. This oxidative stress was accompanied by a progressive decline in intracellular glutathione (GSH) levels, together with reduced Gpx activity and thioredoxin reductase (TrxR) activity (Figure 5I‐K), consistent with weakening of the bacterial antioxidant defense system and redox imbalance associated with ferroptosis. As this imbalance developed, intracellular ATP levels were also significantly reduced (Figure 5H), reflecting subsequent disruption of energy metabolism. Similar trends were observed in *S. aureus* (Figure S13). These findings support that **Dman‐C11 NPs** under NIR irradiation induce ROS‐driven bacterial death accompanied by ferroptosis‐like features [41, 42, 43, 44, 45, 46].

### 2.5 In Vivo Validation of the Antibacterial Activity of Dman‐C11 NPs

The in vivo antibacterial activity of **Dman‐C11 NPs** was further validated in MRSA‐infected wound and keratitis models. An MRSA‐infected open‐wound model was first established to assess antibacterial phototherapy (Figure 6A and Figure S14). Briefly, mice were infected with MRSA and randomly assigned 24 h post‐infection into four groups (*n* = 5 per group): PBS, PBS + laser, **Dman‐C11**, and **Dman‐C11** + laser. Treatment was administered by topical dropwise application onto the infected wound (100 µL, 10 µM) on days 1, 3, and 5, followed by NIR irradiation in the corresponding groups, and wound area was recorded over time. All mice developed pronounced infection by day 3; however, **Dman‐C11** under NIR irradiation markedly accelerated wound closure (Figure 6B). Complete wound healing was observed only in the **Dman‐C11** + laser group by day 13, whereas other groups exhibited significantly delayed repair with 2‐3‐fold larger residual wound areas (Figure S15). Consistently, CFU counts at the infection site were reduced by 20‐fold in the **Dman‐C11** + laser group compared with controls (Figure 6C and Figure S16). Histological evaluation further supported the therapeutic effect. H&E staining revealed reduced inflammatory cell infiltration in the **Dman‐C11** + laser group, while Masson's trichrome staining showed enhanced collagen fiber deposition compared with controls. Immunohistochemical analysis showed decreased CD31 expression in the treated group. Immunofluorescence staining further demonstrated reduced TNF‐α and IL‐1β levels (Figure 6D,E). These results confirmed the therapeutic efficacy of **Dman‐C11 NPs** under NIR irradiation.

**FIGURE 6:**
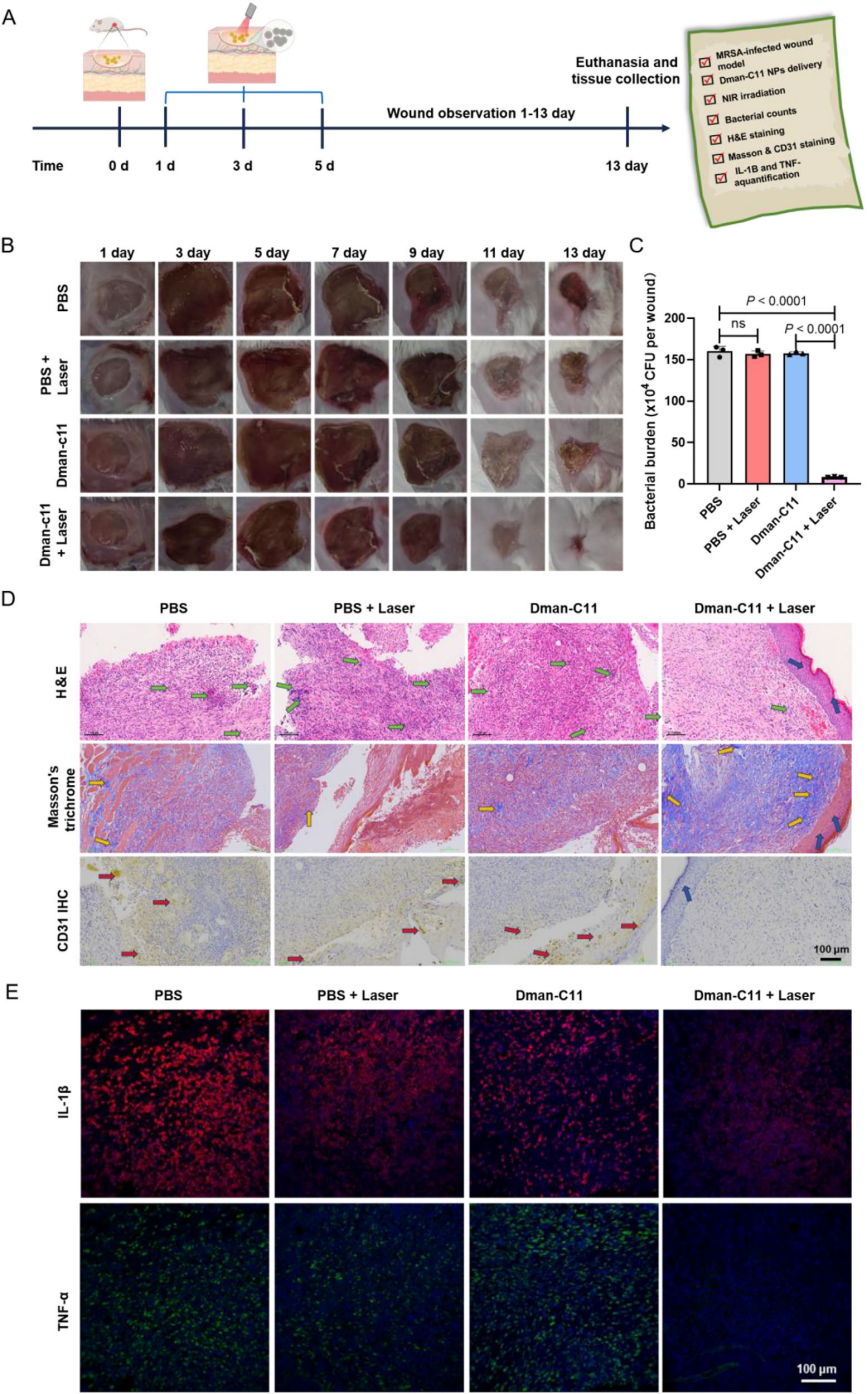
In vivo antibacterial efficacy of **Dman‐C11 NPs** in an MRSA‐infected wound model. (A) Schematic illustration of the treatment and analysis protocol. (B) Representative images of infected wounds under different treatments (*n* = 5 per group). (C) Quantification of MRSA burden in infected skin tissues on day 13. Data are mean ± s.d. (*n* = 3 mice per group). One‐way ANOVA with Tukey's test. (D) H&E and Masson's trichrome staining of infected wounds, together with CD31 immunohistochemical analysis after different treatments. Scale bar, 100 µm. (E) Immunofluorescence staining of TNF‐α and IL‐1β following different treatments. Green, blue, yellow, and red arrowheads indicate inflammatory cell infiltration, epidermis, collagen fibers, and CD31‐positive microvessels respectively.

Given the potent antibacterial efficacy of **Dman‐C11 NPs** in the MRSA‐infected wound model, we next evaluated its therapeutic potential in bacterial keratitis (BK), a severe MRSA‐associated ocular infection that can lead to visual impairment if untreated, where photodynamic antimicrobial therapies has been used [47, 48]. An MRSA‐induced keratitis model was established by applying 10 µL of bacterial suspension (1.0 × 10^7^ CFU/mL) onto mechanically injured corneas of mice. After 24 h of infection, animals were randomly assigned to five treatment groups, with ofloxacin and PBS serving as positive and negative controls, respectively. Imaging obtained using an ophthalmic surgical microscope showed that **Dman‐C11** combined with NIR irradiation markedly alleviated corneal opacity and inflammation, exhibiting a therapeutic efficacy comparable to that of ofloxacin and resulting in a clearer corneal surface by day 7 (Figure 7A,B and Figure S17). Consistently, CFU counting revealed a ∼15‐fold reduction in bacterial burden in the **Dman‐C11** + laser group compared with controls, in agreement with clinical scoring (Figure 7C,D and Figure S18). Histological analysis (H&E and Masson's trichrome staining) further confirmed preservation of corneal architecture and reduced tissue damage in the treated group, comparable to that observed in the antibiotic‐treated group (Figure 7E). These results indicate that **Dman‐C11**‐mediated aPDT effectively reduces bacterial burden and preserves corneal structure in MRSA‐induced keratitis.

**FIGURE 7:**
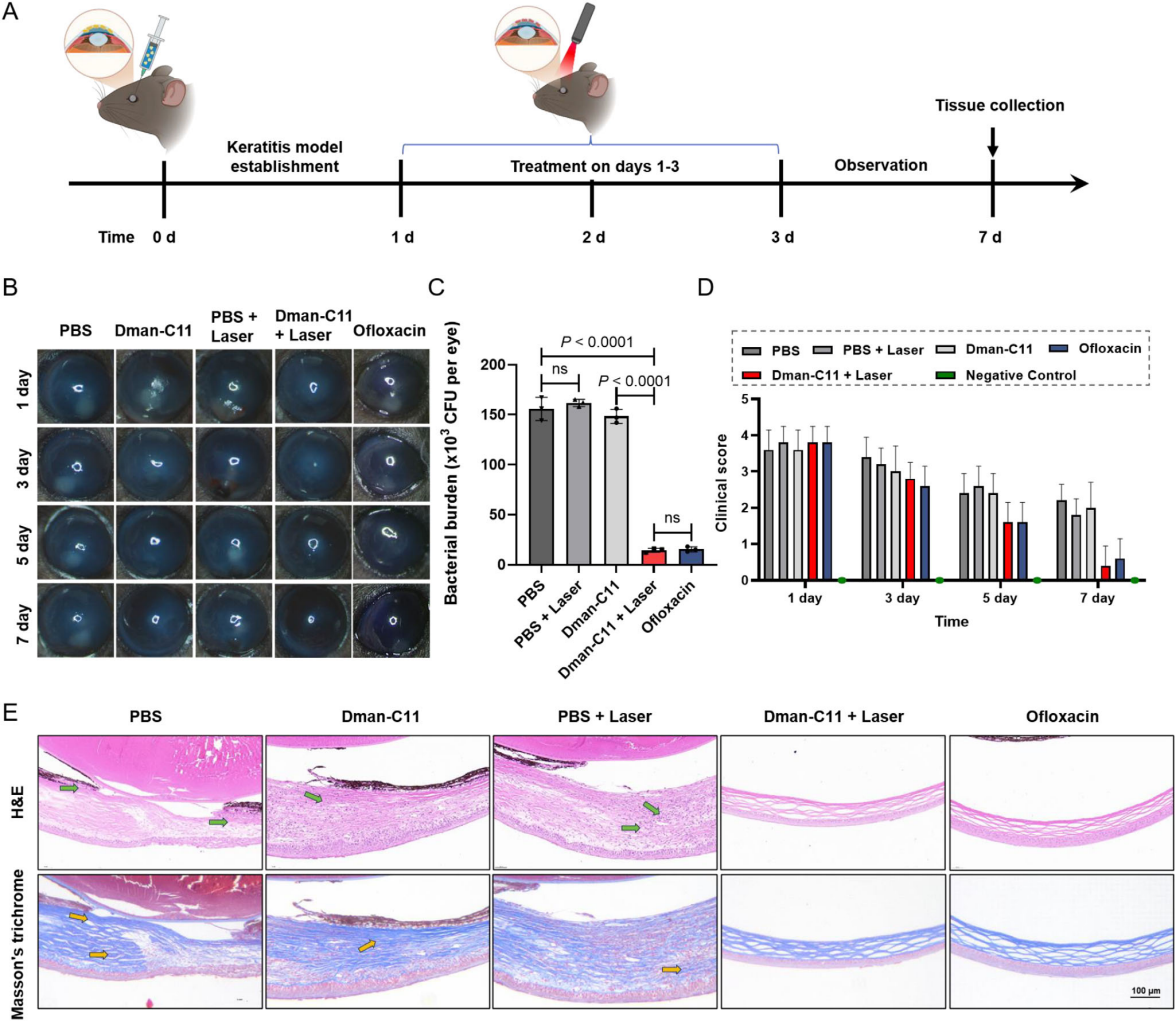
In vivo evaluation of the antibacterial activity of **Dman‐C11 NPs** in a bacterial keratitis model. (A) Schematic illustration of the experimental design for the infectious keratitis model induced by MRSA. (B) Representative images of mouse corneas under different treatments, obtained using an ophthalmic surgical microscope (*n* = 4 per group). (C) CFU counts in infected eyes following treatment. Data are mean ± s.d. (*n* = 3 mice per group). One‐way ANOVA with Tukey's test. (D) Clinical scoring of corneal severity based on opacity area, opacity density, and surface regularity. (E) Representative H&E‐ and Masson's trichrome‐stained corneal sections collected on day 7 after different treatments. Green arrowheads indicate inflammatory cell infiltration, and yellow arrowheads indicate disorganized stromal collagen fibers. Representative images from three mice per group are shown. Scale bar, 100 µm.

## 3 Conclusions

In conclusion, we have developed an immune‐recruiting amphiphilic hemicyanine‐based molecule, **Dman‐C11**, that exploits bacterial membrane‐triggered supramolecular disassembly to regulate antibacterial function. **Dman‐C11** spontaneously assembles under physiological conditions into nanoparticles with aggregation‐caused fluorescence quenching and suppressed basal ROS generation. Upon association with MRSA membranes, supramolecular disassembly redistributes the probe within the bacterial membrane, restoring fluorescence for high‐contrast bacterial imaging and activating photodynamic activity. Under NIR irradiation, **Dman‐C11** generates efficient singlet oxygen, inducing ferroptosis‐like bacterial death characterized by extensive lipid peroxidation and oxidative membrane damage. Meanwhile, the D‐mannosamine motif promotes immune‐cell recruitment toward bacterial pathogens, thereby enhancing host‐assisted bacterial clearance. This work establishes a supramolecularly regulated single‐molecule theranostic strategy in which bacterial membrane‐triggered structural reorganization controls imaging activation, photodynamic antibacterial activity, and immune‐assisted clearance, enabling effective treatment of MRSA infections in vivo.

## 4 Statistical Analysis

Data are presented as the mean ± standard deviation (SD) unless otherwise indicated. Detailed data preprocessing, sample sizes (*n*), and definitions of independent replicates are provided in the corresponding figure legends or experimental methods. Comparisons between two independent groups were performed using an unpaired, two‐sided Student's *t*‐test. Comparisons among more than two groups, including comparisons among treatment groups at individual time points, were performed using one‐way ANOVA followed by Tukey's multiple‐comparison test. Experiments involving two independent factors were analyzed using two‐way ANOVA followed by Bonferroni's multiple‐comparison test. Fisher's exact test with Benjamini–Hochberg correction was used for enrichment analysis. The significance threshold was set at α = 0.05 (*P* < 0.05); for enrichment analysis, Benjamini–Hochberg‐adjusted *Q* values < 0.05 were considered significant. Statistical significance is indicated by exact *P* values or by the following symbols: ns, not significant; *P* < 0.05; *P* < 0.01; *P* < 0.001; and *P* < 0.0001. Unless otherwise indicated, statistical analyses were performed using GraphPad Prism 11.0.1.

The authors have cited additional references within the Supporting Information [49].

## Author Contributions

**Jiacheng Du**: writing – review and editing, investigation, validation, data curation, visualization, writing – original draft, methodology, formal analysis. **Yongyi Chen**: methodology, investigation, writing – original draft, visualization, data curation, formal analysis. **Ling Lei**: investigation. **Yunzhe Tao**: visualization. **Yu Peng**: validation, formal analysis, software. **Qingyang Zhang**: project administration, supervision. **Qinghua Wang**: project administration, supervision, methodology, writing – review and editing. **Hai‐Yu Hu**: conceptualization, writing – review and editing, project administration, supervision, funding acquisition, writing – original draft.

## Ethics Statement

All experiments involving pathogenic microorganisms were performed in Biosafety Level 2 (BSL‐2) laboratory facilities in accordance with institutional safety protocols. All animal studies were conducted in accordance with national and provincial regulations governing the care and use of laboratory animals. The experimental protocols were reviewed and approved by the Animal Care and Use Committee of the Institute of Materia Medica, Chinese Academy of Medical Sciences (Approval Nos. 00003784 and 00006355).

## Conflicts of Interest

The authors declare no conflicts of interest.

## Supporting information




**Supporting File**: advs78522‐sup‐0001‐SuppMat.pdf.

## Data Availability

The data that support the findings of this study are available from the corresponding author upon reasonable request.
