# Supplementary material for: MRSA‐Triggered Disassembly of an Immune‐Recruiting NIR Probe for Bacterial Imaging and Clearance
Source: Adv Sci (Weinh). 2026 Oct 31:e78522. Online ahead of print. doi: 10.1002/advs.78522 (PMC13723719; doi:10.1002/advs.78522)
Supplement: Supplementary file 1 [file ADVS-9999-e78522-s001.pdf]

Supporting Information

MRSA-Triggered Disassembly of an Immune-Recruiting NIR Probe for Bacterial Imaging and Clearance

Jiacheng Du†, Yongyi Chen†, Ling Lei, Yunzhe Tao, Yu Peng, Qingyang Zhang, Qinghua Wang, and Hai-Yu Hu**

†These authors contributed equally to this work.
*Corresponding authors.

Table of Contents

Table of Contents S2

Experimental Procedures S3

1. Materials and Characterizations S3

2. Synthesis procedure S3

3. Method S5

3.1. Molecular Dynamics Simulation S5

3.2. Spectral Characterization of **Dman-C11** S6

3.3. Particle Size and Zeta Potential Measurements S6

3.4. Biocompatibility Assessment of **Dman-C11 NPs** S6

3.5. Antibacterial Properties of **Dman-C11 NPs** S7

3.6. Antibacterial Mechanism of **Dman-C11 NPs** S8

3.7. Cellular and Bacterial Imaging S9

3.8. The Immune Recognition Labeling of Bacteria by **Dman-C11 NPs** S9

3.9. In vivo Imaging in Mice S10

3.10. Antibacterial Activity of **Dman-C11 NPs** in vivo S10

3.11. Flow Cytometry Analysis S11

Results and Discussion S12

NMR and LC-MS Spectrum S19

Reference S25

Experimental Procedures

1. Materials and Characterizations

All reagents and solvents were commercially available and used without further purification unless otherwise noted. Four bacterial strains *Staphylococcus aureus* (*S. aureus*, ATCC 29213), Methicillin-resistant *Staphylococcus aureus* (MRSA, ATCC 33592), *Klebsiella pneumoniae* (*K. pneumoniae*, ATCC 700603), and *Escherichia coli* (*E.coli*, ATCC 25922) were purchased from American Type Culture Collection (ATCC), USA. Nuclear magnetic resonance (NMR) spectra were recorded on Q.One Instruments Ltd or Bruker at 400, 500, or 700 MHz. High-resolution mass spectra (HRMS) were measured with Thermo Exactive Plus Obitrap mass spectrometer with electrospray ionization. UV-Vis absorption and fluorescence emission spectra were recorded using a Tecan Spark 10M multimode microplate reader. Confocal laser scanning microscope (CLSM) images were performed on Leica TCS SP8 X confocal microscope and ISS Q2 confocal laser scanning system. Rabbit red blood cells were purchased from Shanghai yuanye Bio-Technology Co., Ltd. Liperfluo was purchased from Dojindo Laboratories (Kumamoto, Japan). ATP, GSH, TrxR assay kits were procured from Beyotime Biotechnology (Shanghai, China). *In vivo* imaging was obtained on the IVIS Spectrum CT system (PerkinElmer, USA).

2. Synthesis procedure

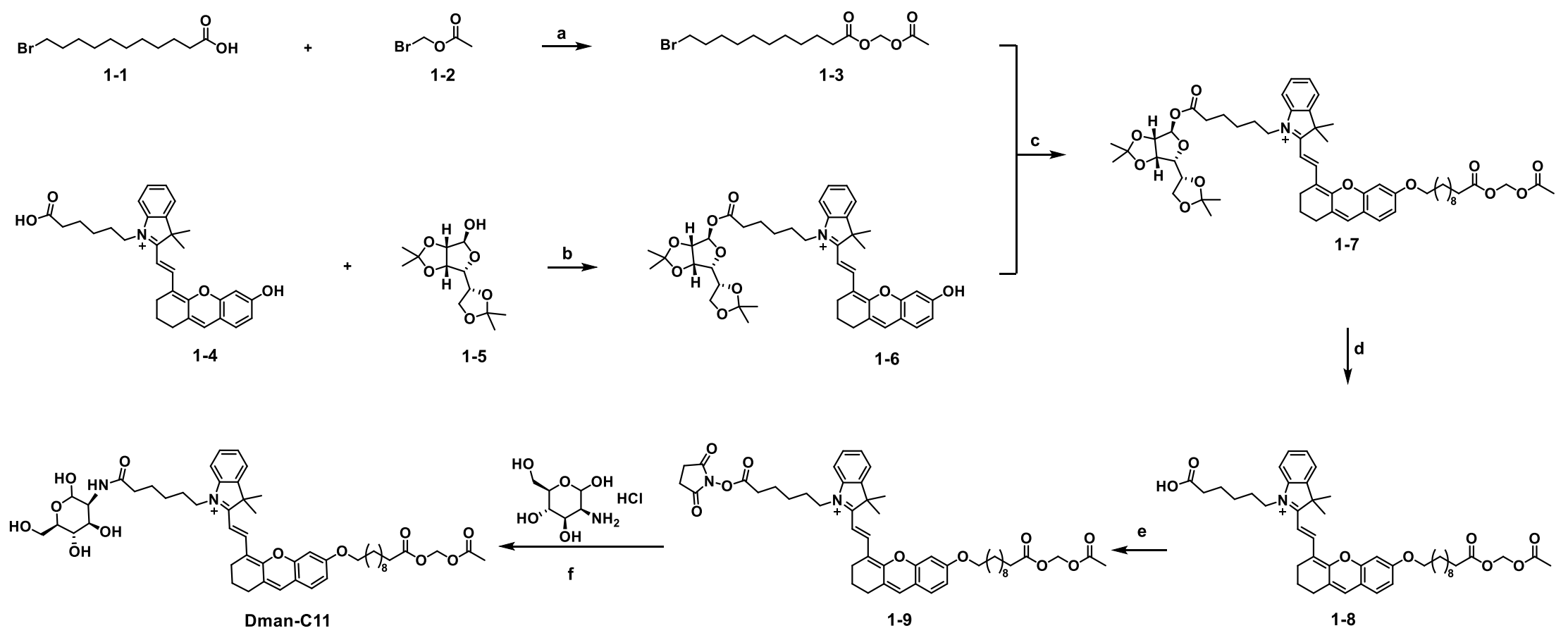

Scheme S1. Synthetic procedure for the probe **Dman-C11.**

Reagent and condition:(a) DIPEA, ACN, 50 ºC, 78.7 %; (b) EDCI, DMAP, DCM/DMF, 37.4 %; (c) DIPEA, DMF, 65 ºC, 25.8 %; (d) 90 % TFA/H2O, 0 ºC; (e) DSC, TEA, DMF, r.t.; (f) TEA, DMF, r.t., 26.3 %.

Synthesis of Compound 1-3

11-Bromoundecanoic acid 1-1 (1.000 g, 3.77 mmol) was dissolved in anhydrous acetonitrile (6.0 mL) under an argon atmosphere at room temperature. Bromomethyl acetate 1-2 (1.313 mL, 7.54 mmol) was added dropwise into the solution, followed by the addition of *N*, *N'*-diisopropylethylamine (740 µL, 7.54 mmol). The reaction mixture was stirred at 50 ºC for 1 hour.

The reaction mixture was directly purified by silica gel column chromatography (1% ethyl acetate/petroleum ether) to afford compound 1-3 as a colorless oil (1.001 g, 78.7% yield). ^{1}H NMR (400 MHz, Chloroform-*d*) δ 5.73 (s, 1H), 3.40 (t, *J* = 6.8 Hz, 2H), 2.35 (t, *J* = 7.5 Hz, 2H), 2.10 (s, 3H), 1.84 (p, *J* = 7.0 Hz, 2H), 1.62 (p, *J* = 7.7 Hz, 2H), 1.40 (q, *J* = 7.1 Hz, 2H), 1.28 (s, 10H). ^{13}C NMR (100 MHz, Chloroform-*d*) δ 172.52, 169.70, 79.12, 34.02, 33.93, 32.81, 29.33, 29.28, 29.14, 28.95, 28.71, 28.14, 24.55, 20.75.

Synthesis of Compound 1-6

Compound hemicyanine 1-4 [49](1.200 g, 2.47 mmol), (3aS ,4S, 6R,6aS) -6- ((R)-2,2-dimethyl-1,3-dioxolan-4-yl) -2,2-dimethyltetrahydrofuro[3,4-d][1,3]dioxol-4-ol 1-5 (1.299 g, 2.06 mmol), 4-(dimethylamino) pyridine (DMAP, 0.43g, 2.31mmol), 1-ethyl-3-(3-dimethylaminopropyl) carbodiimide (EDCI, 0.706 g, 1.54 mmol), and a mixture of anhydrous dichloromethane and anhydrous *N*,*N'*-diisopropylethylamine (5:1, 12 mL) were added to the reaction system. The mixture was stirred at room temperature for approximately 1 hour. After completion, the reaction mixture was diluted with dichloromethane, washed with saturated brine for three times, dried over by anhydrous sodium sulfate, filtered, and concentrated under reduced pressure. The residue was purified by silica gel column chromatography (4% methanol/dichloromethane) to afford compound 1-6 as a blue solid (0.642 g, 37.4% yield). ^{1}H NMR (500 MHz, Methanol-d_4) δ 8.65 (d, *J* = 14.6 Hz, 1H), 7.60 (d, *J* = 7.5 Hz, 1H), 7.52 – 7.34 (m, 5H), 6.82 (dd, *J* = 8.6, 2.2 Hz, 1H), 6.77 (d, *J* = 2.3 Hz, 1H), 6.34 (d, *J* = 14.7 Hz, 1H), 6.01 (s, 1H), 4.82 (dd, *J* = 5.8, 3.5 Hz, 2H), 4.68 (d, *J* = 5.8 Hz, 1H), 4.33 (q, *J* = 6.0 Hz, 1H), 4.26 (t, *J* = 7.4 Hz, 2H), 4.07 (dd, *J* = 6.4, 3.5 Hz, 1H), 4.04 – 3.99 (m, 1H), 3.89 (dd, *J* = 8.6, 5.5 Hz, 1H), 2.76 (t, *J* = 5.7 Hz, 2H), 2.70 (t, *J* = 6.0 Hz, 2H), 2.38 (t, *J* = 7.2 Hz, 2H), 1.91 (dt, *J* = 21.9, 6.8 Hz, 4H), 1.79 (s, 6H), 1.71 (p, *J* = 7.2 Hz, 2H), 1.54 – 1.46 (m, 2H), 1.41 (s, 3H), 1.35 (s, 3H), 1.29 (d, *J* = 2.9 Hz, 6H). ^{13}C NMR (100 MHz, Methanol-d_4) δ 176.64, 172.09, 164.18, 162.76, 155.04, 144.80, 141.77, 141.69, 135.40, 129.15, 128.79, 126.52, 125.80, 122.38, 115.07, 114.33, 112.72, 112.07, 108.69, 102.22, 101.65, 100.63, 84.86, 82.13, 79.25, 72.95, 65.99, 50.24, 44.31, 33.16, 28.53, 27.14, 26.89, 25.74, 25.59, 24.81, 23.99, 23.85, 23.72, 23.29, 20.35.

Synthesis of Compound 1-7

Compound 1-6 (0.100 g, 0.137 mmol), compound 1-3 (0.139 g, 0.411 mmol), *N*,*N'*-diisopropylethylamine (96 μL, 0.548 mmol), and anhydrous dimethylformamide (2 mL) were added to the reaction system. The mixture was heated to 60 ºC and stirred overnight. After completion, the reaction mixture was diluted with dichloromethane, washed with saturated brine, dried over by anhydrous sodium sulfate, filtered, and concentrated under reduced pressure. The residue was purified by silica gel column chromatography (2% methanol/dichloromethane) to afford compound 1-7 as a blue solid (0.037 g, 28.5% yield). ^{1}H NMR (400 MHz, Methanol-d_4) δ 8.79 (d, *J* = 14.8 Hz, 1H), 7.68 (d, *J* = 7.4 Hz, 1H), 7.57 (d, *J* = 6.0 Hz, 2H), 7.52 – 7.43 (m, 3H), 7.05 (d, *J* = 2.2 Hz, 1H), 7.00 (dd, *J* = 8.6, 2.3 Hz, 1H), 6.53 (d, *J* = 14.9 Hz, 1H), 6.05 (s, 1H), 5.74 (s, 2H), 4.85 (d, *J* = 3.5 Hz, 1H), 4.71 (d, *J* = 5.9 Hz, 1H), 4.38 (p, *J* = 6.5 Hz, 3H), 4.17 (t, *J* = 6.3 Hz, 2H), 4.11 (dd, *J* = 6.4, 3.5 Hz, 1H), 4.05 (dd, *J* = 8.5, 6.4 Hz, 1H), 3.92 (dd, *J* = 8.6, 5.5 Hz, 1H), 2.81 (t, *J* = 6.0 Hz, 2H), 2.76 (t, *J* = 6.0 Hz, 2H), 2.40 (dt, *J* = 14.9, 7.2 Hz, 4H), 2.09 (s, 3H), 1.97 (q, *J* = 8.0, 7.4 Hz, 4H), 1.86 (s, 6H), 1.75 (m, 4H), 1.66 – 1.61 (m, 2H), 1.57 – 1.52 (m,

4H), 1.44 (s, 3H), 1.39 (s,3H), 1.36 (s, 9H), 1.32 (d, J = 3.4 Hz, 6H).^{13}C NMR (176 MHz, Methanol-d_4) δ 177.59, 172.38, 172.06, 169.70, 162.93, 161.91, 154.58, 145.53, 142.08, 141.55, 134.16, 128.87, 127.06, 122.43, 115.76, 114.41, 113.55, 112.71, 112.48, 108.68, 103.23, 100.77, 100.63, 84.86, 82.14, 79.25, 79.01, 72.95, 68.65, 66.00, 53.46, 50.57, 44.57, 33.26, 33.15, 29.20, 29.08, 29.03, 28.93, 28.83, 28.62, 27.03, 25.69, 25.61, 24.83, 24.31, 24.01, 23.84, 23.31, 20.28, 19.20.

Synthesis of **Dman-C11**

Compound 1-7 was added to a pre-cooled mixture of trifluoroacetic acid and water (9:1, 1 mL). After stirring for approximately 10 minutes, the mixture was diluted with dichloromethane, and water was added. The organic layer was extracted, dried, and concentrated. *N*,*N*'-disuccinimidyl carbonate (DSC, 0.010 g, 0.037 mmol), anhydrous dichloromethane (2.0 mL), and triethylamine (10 μL) were added to the residue. After stirring for 10 minutes, the reaction progress was monitored by TLC. Upon completion, the mixture was concentrated, and residual DSC was removed using a mixture of ethyl acetate and petroleum ether. After drying for about 3 hours, the residue was then combined with mannose hydrochloride (0.040 g, 0.188 mmol), anhydrous *N*,*N*'-diisopropylethylamine (2 mL), and triethylamine (15 μL). The mixture was stirred at room temperature for 45 minutes. After completion, the reaction mixture was diluted with dichloromethane, washed with saturated brine, dried over by anhydrous sodium sulfate, filtered, and concentrated under reduced pressure. The residue was purified by silica gel column chromatography (2% methanol/dichloromethane) to afford **Dman-C11** as a blue solid (0.009 g, 26.3% yield). ^{1}H NMR (700 MHz, Methanol-d_4) δ 8.77 (d, J = 14.8 Hz, 1H), 7.65 (d, J = 7.5 Hz, 1H), 7.54 (dt, J = 11.2, 7.5 Hz, 2H), 7.49 – 7.40 (m, 3H), 7.03 (d, J = 2.4 Hz, 1H), 7.00 – 6.97 (m, 1H), 6.50 (d, J = 14.8 Hz, 1H), 5.71 (s, 2H), 4.35 (q, J = 7.5 Hz, 2H), 4.28 (dd, J = 4.6, 1.6 Hz, 1H), 4.14 (t, J = 6.3 Hz, 2H), 4.00 (dd, J = 9.7, 4.7 Hz, 2H), 3.89 – 3.81 (m, 1H), 3.78 – 3.73 (m, 2H), 3.65 (dt, J = 9.4, 4.7 Hz, 1H), 3.57 (t, J = 9.8 Hz, 1H), 2.79 (t, J = 6.0 Hz, 2H), 2.73 (t, J = 6.2 Hz, 2H), 2.35 (t, J = 7.4 Hz, 3H), 2.34 – 2.25 (m, 2H), 2.06 (s, 3H), 1.93 (dq, J = 24.3, 7.6, 6.9 Hz, 4H), 1.83 (s, 8H), 1.73 (dq, J = 15.0, 7.4 Hz, 2H), 1.61 (t, J = 7.2 Hz, 2H), 1.53 (ddt, J = 12.1, 7.6, 2.5 Hz, 3H), 1.44 – 1.39 (m, 2H), 1.33 (s, 9H).^{13}C NMR (176 MHz, Methanol-d_4) δ 177.54, 175.03, 172.41, 169.72, 162.93, 161.91, 154.59, 154.57, 145.57, 142.08, 141.57, 134.07, 128.76, 126.92, 122.37, 122.34, 115.75, 114.39, 113.51, 112.49, 103.20, 100.72, 93.63, 78.99, 72.01, 69.23, 68.61, 67.05, 60.73, 53.59, 50.54, 44.56, 35.03, 33.24, 29.18, 29.06, 29.00, 28.91, 28.81, 28.69, 28.60, 27.10, 26.99, 26.97, 25.95, 25.67, 24.98, 24.30, 23.75, 20.29, 19.16.

3. Method

3.1. Molecular Dynamics Simulation

The molecular dynamics simulations were performed using GROMACS 5.15. Twenty structurally optimized **Dman-C11** molecules were randomly distributed in a cubic box (8×8×8 nm^3) filled with SPC/E water molecules. The system first underwent energy minimization (EM) using the steepest descent algorithm. Subsequently, a 50 ps equilibration was conducted in the NVT ensemble at 300 K. Finally, production molecular dynamics was carried out for 50 ns in the NPT ensemble (1 atm, 300 K) with periodic boundary conditions applied in all directions.

3.2. Spectral Characterization of **Dman-C11**

The probe **Dman-C11** was initially dissolved in DMSO to prepare a 1 mM stock solution, which was stored at -20 ºC in the dark for further use. Prior to experiments, the stock solution was diluted with PBS to achieve a final concentration of 10 μM. The slit width for all measurements was set to 5.0 nm, and the excitation wavelength was fixed at 600 nm. Unless otherwise specified, all solutions were prepared and measured immediately.

Validation of Hydrophobic Interactions

Dman-C11 was dissolved in PBS buffer to prepare a stock solution. To validate that hydrophobic interactions are the primary driving force for self-assembly, the disassembly of **Dman-C11 NPs** was induced by the organic solvent 1,4-dioxane and the non-ionic surfactant Tween 20, respectively. For the 1,4-dioxane-induced disassembly assay, the **Dman-C11** stock solution was mixed with different volumes of 1,4-dioxane to reach final volume fractions of 10%, 20%, 30%, 40%, 50%, 60%, 70%, 80%, 90%, and 95% (v/v), with PBS alone as the control. After incubation in the dark for 1 min, the fluorescence intensity of each group was measured using a microplate reader and photographed (Figure B). For the Tween 20-induced disassembly assay, the **Dman-C11** stock solution was mixed with different volumes of Tween 20 to reach final concentrations of 0.5‰, 1‰, 1.5‰, 2‰, 3‰, 4‰, and 5‰ (v/v), with PBS alone as the control. After incubation in the dark for the same period, the fluorescence intensity of each group was measured.

3.3. Particle Size and Zeta Potential Measurements

Particle Size Measurement

The probe **Dman-C11** was prepared as a 10 μM solution (200 μL total volume) and transferred to a sample cell. Dynamic light scattering (DLS) measurements were performed using standard instrument parameters.

Zeta Potential Measurement

The probe **Dman-C11** was diluted by ddH_2O and prepared as a 10 μM solution (1000 μL total volume) and transferred to a sample cell. Zeta potential measurements were conducted using standard instrument parameters.

3.4. Biocompatibility Assessment of **Dman-C11 NPs**

Cytotoxicity Assay

The cytotoxicity of **Dman-C11 NPs** was evaluated using the standard CCK-8 assay. Briefly, HepG2, Malme-3M, 4T1, and BEAS-2B cells were seeded in 96-well flat-bottom microplates at a density of 1×10^4 cells/mL (100 μL per well) and incubated at 37 ºC for 24 hours. Subsequently, the cells were treated with varying concentrations of **Dman-C11 NPs** (0–20 μM) and incubated for an additional 24 hours. After incubation, the medium was removed, and 110 μL of CCK-8 working solution (10 μL CCK-8 reagent + 100 μL fresh medium) was added to each well. The

plates were incubated at 37 ºC for 45–60 minutes, and the absorbance was measured at 450 nm using a Tecan Spark 10M microplate reader. Cell viability was calculated using the formula:

Cell viability (%) = $(A_A-A_0)/(A_s-A_0) \times 100$

where A_A is the absorbance of the test group, A_s is the absorbance of the control group, and A_0 is the absorbance of the blank group (no cells). All experiments were performed in triplicate to ensure reproducibility.

Hemolysis Assay

Rabbit red blood cells (RBCs) were centrifuged at 8000–8500 rpm for 3 minutes, and the supernatant was carefully removed. The pelleted RBCs were washed with PBS and centrifuged repeatedly until the supernatant was colorless. The washed RBCs were resuspended in PBS to prepare a 4% RBC suspension. For the assay, the negative control was PBS alone, and the positive control was ddH_2O. For the test groups, RBCs were treated with varying concentrations of **Dman-C11 NPs** (1.56, 3.13, 6.25, 12.5, and 25 μM) and incubated for 30 minutes. After centrifugation, 100 μL of the supernatant was transferred to a 96-well plate, and the absorbance was measured at 540 nm. The hemolysis rate was calculated using the formula:

Hemolysis rate (%) = $(A_A-A_0)/(A_s-A_0) \times 100$

where A_A is the absorbance of the test group, A_s is the absorbance of the control group, and A_0 is the absorbance of the blank group (no cells). All experiments were performed in triplicate to ensure reproducibility.

3.5. Antibacterial Properties of **Dman-C11 NPs**

Minimum Inhibitory Concentration (MIC) Assay

Test bacteria were cultured in 20 mL of medium at 37 ºC overnight. The bacterial suspension was diluted to $OD_{600} = 5 \times 10^{-4}$, and 200 μL was added to the first column of a 96-well plate. **Dman-C11 NPs** was added to the first well at a final concentration of 50 μM, and serial two-fold dilutions were performed across the plate. The plate was incubated at 37 ºC for 16 hours, and the MIC was determined as the lowest concentration of **Dman-C11 NPs** that inhibited visible bacterial growth.

Reactive Oxygen Species (ROS) Generation Assay

Dman-C11 NPs (10 μM) was mixed with a singlet oxygen fluorescent probe (9,10-Anthracenediyl-bis(methylene)dimalonic acid, ABDA) in a 96-well plate. The absorption spectrum (300–450 nm) was recorded before and after irradiation with a 655 nm laser (100 mW/cm²) for 1, 2, 3, 4, 5, and 10 minutes.

Bacterial Killing Assay

S.aureus (ATCC 29213 and ATCC 33592) were treated with **Dman-C11 NPs** (0.2, 1.0, and 5.0 μM) under three conditions: (1) probe incubation without laser irradiation, (2) probe incubation followed by 655 nm laser irradiation (200 mW/cm², 15 minutes), and (3) untreated control. After treatment, the bacterial suspensions were diluted 10^{-6}-fold, and 100 μL was spread on agar plates. The plates were incubated at 37 ºC overnight, and bacterial growth was analyzed the next day.

3.6. Antibacterial Mechanism of **Dman-C11 NPs**

Cytoplasmic Membrane Depolarization Assay

Mid-log phase MRSA cultures were harvested by centrifugation, washed, and resuspended in PBS to a density of 10^8 CFU/mL. The bacterial suspension was divided into groups, and **Dman-C11** was added at different final concentrations (0, 2.5, 5, and 10 μM). The 0 μM group received laser irradiation only, while the Blank and **Dman-C11** alone groups served as negative controls without laser irradiation. NIR irradiation was performed at 655 nm with a power density of 200 mW cm^{-2}. Subsequently, 1 μM $DiBAC_4(3)$ was added, and the samples were incubated in the dark for 15 min. The fluorescence intensity of $DiBAC_4(3)$ was measured using a fluorescence microplate reader at the appropriate excitation/emission wavelengths. All experiments were performed in triplicate independently.

Detection of Protein Leakage

Bacterial cell membrane damage in *S.aureus* and MRSA was measured using the BCA kit. Briefly, after the corresponding antibacterial treatment, the mixture was collected and centrifuged, and the supernatant was added to the BCA working solution and incubated at 37 ºC for 30 min. Subsequently, the leakage of protein within the bacteria was detected by measuring the optical density value at 562 nm via a Microplate Reader.

Evaluation of Ferroptosis Factors at the Cellular Level

MRSA (ATCC 33592) was cultured overnight for 12 hours in 200 mL of TSB medium. *S.aureus* was cultured overnight for 12 h in 200 mL of LB medium. The bacteria were separated by centrifugation and washed twice with PBS buffer. The experiment was divided into six groups (OD_{600} = 10; 655 nm laser, 200 mW/cm^2, 10 min): Bacteria; Bacteria + 5 μM **Dman-C11**; Bacteria + 15 μM **Dman-C11**; Bacteria+Laser; Bacteria + 5 μM **Dman-C11**+Laser; Bacteria + 15 μM **Dman-C11**+Laser. The supernatant from each group was collected and centrifuged at 10,000 rpm for 10 minutes at 4 ºC. And then follow the guidelines of the kit.

Bacterial RNA Sequencing (RNA-seq)

MRSA was cultured to OD_{600} = 5, centrifuged, and washed twice with PBS. The bacterial pellets were resuspended in PBS and treated with **Dman-C11 NPs** (10 μM). The suspensions were divided into two groups: (1) irradiated with a 655 nm laser (200 mW/cm², 20 minutes) and (2) non-irradiated control. After treatment, the bacteria were collected, centrifuged, and flash-frozen in liquid nitrogen for RNA-seq analysis by BGI Genomics.

RNA Sequencing and Bioinformatic Analysis

RNA-seq was performed using three biological replicates per group. Clean reads were aligned to the reference genome using Bowtie2 (v2.2.5). Gene expression was quantified using RSEM (v1.2.8). Differential expression analysis between the control and **Dman-C11** NP-treated groups was performed using DESeq2. Genes with $|\log_2 FC| > 1$ and Benjamini–Hochberg-adjusted *Q* values < 0.05 were defined as differentially expressed genes (DEGs).

Functional enrichment analyses of DEGs were performed against the Kyoto Encyclopedia of Genes and Genomes (KEGG) and Gene Ontology (GO) databases. KEGG enrichment was analyzed using the phyper function in R, with *P* values adjusted by the Benjamini–Hochberg method. GO enrichment was performed using the TermFinder package. Terms with adjusted *Q* values < 0.05 were considered significantly enriched. Heatmaps were generated using the pheatmap package in R based on normalized gene-expression values, with row-scaled Z-scores displayed.

3.7. Cellular and Bacterial Imaging

Cellular Imaging

HK-2 cells and HepG2 cells were seeded in 8-well plates at a density of 1×10^4 cells/mL and incubated overnight. The cells were treated with **Dman-C11 NPs** (1 μM) in phenol red-free DMEM/F_{12} medium for 30 minutes at 37 ºC. After washing with PBS, the cells were stained with Hoechst 33342 (1‰) for 15 min and imaged using a Leica TCS SP8 X confocal microscope (63 × oil immersion lens, excitation: 600 nm, emission: 630–750 nm).

Bacterial Imaging

Bacterial strains (*S.aureus*, MRSA, *K.pneumoniae* ATCC 700603, *P.aeruginosa* ATCC 27853, *E.coli* ATCC 25922) were cultured overnight, washed with PBS, and resuspended to OD_{600} = 1.0. The bacteria were treated with **Dman-C11 NPs** (1 μM) for 10 minutes at 37 ºC, stained with Hoechst 33342 (1‰), and imaged using a Leica TCS SP8 X confocal microscope (63× oil immersion lens).

Localization Experiment

Confocal fluorescence images were captured on an ISS Q2 confocal laser scanning system coupled with a Nikon TE2000 microscope with a 60×/1.2 NA WI objective lens. To stain the membrane, 5 μM of **Dman-C11 NPs** was added in the suspension of *S. aurues* in PBS (1% DMSO), with an OD_{600} of 0.5~1.0. After incubation at 37 ºC for 0.5 h, 1~2 μL cells were sampled directly and mounted on microscope slides covered with a thin film of 1% agarose in water for imaging. Images were acquired with ISS VistaVision and processed by ImageJ. λ_{ex} = 640 nm, λ_{em} = 663-733 nm.

3.8. The Immune Recognition Labeling of Bacteria by **Dman-C11 NPs**

To evaluate the phagocytic efficiency of RAW264.7 macrophages against *S. aureus*, we first conducted the culture and pretreatment of RAW264.7 cells following the protocol described below: BALB/c mouse tibias subjected to the designated treatment were harvested, and the bone marrow was flushed out with high-glucose medium to prepare a bone marrow suspension. The bone marrow suspension was filtered through a 0.22-μm filter to culture RAW264.7 cells for 24 h. Subsequently, the culture medium was replaced with an antibiotic-free medium. The enhanced green fluorescent protein (EGFP)-expressing *S. aureus* and EGFP-expressing *S. aureus* labeled with **Dman-C11 NPs** were added to the macrophage culture wells for the control group and experimental group. For the D-mannosamine-blocked group, macrophages were pretreated with 50 μM D-mannosamine for 1 hour prior to the addition of the same EGFP-expressing *S. aureus* labeled with **Dman-C11 NPs** to the culture system. After 2 h of co-incubation, the macrophages were rinsed three times with phosphate-buffered saline (PBS) to eliminate residual non-internalized bacteria in the supernatant. Finally, flow cytometry and fluorescence staining assays were conducted to determine and quantify the capacity of **Dman-C11 NPs** to modulate macrophage recruitment and phagocytic activity.

3.9. *In vivo* Imaging in Mice

All animal experiments were approved by the Institutional Animal Care and Use Committee of the Chinese Academy of Medical Sciences and Peking Union Medical College (approval number: 00003784) and conducted in accordance with institutional guidelines.

4T1 Breast Cancer Model

A total of 5×10^6 4T1 breast cancer cells in 100 μL PBS were subcutaneously implanted into the right tight of each female BALB/c mouse (6–8 weeks). MRSA (OD_{600} = 2, 100 μL) was subcutaneously injected into the right hind leg 18 hours prior to imaging. **Dman-C11 NPs** (25 μM, 100 μL) was administered via tail vein injection, and *in vivo* fluorescence imaging was performed using animal imaging system (excitation: 640 nm, emission: 680 nm). After imaging, the mice were euthanized, and major organs (heart, liver, spleen, lungs, kidneys) and infected tissues were excised for ex vivo imaging to evaluate probe distribution and metabolism.

Mouse Model of Lipopolysaccharides (LPS)-Induced Acute Dermatitis

BALB/c-nu mice were acclimatized for one week before experiments. MRSA (OD_{600} = 2, 100 μL) was subcutaneously injected into the right hind leg 18 h prior to imaging, while LPS (9 mg/kg in saline) was injected into the left hind leg 6 hours prior to imaging. **Dman-C11 NPs** (25 μM, 100 μL) was administered via tail vein injection, and *in vivo* fluorescence imaging was performed using animal imaging system (excitation: 640 nm, emission: 680 nm). After imaging, the mice were euthanized, and major organs (heart, liver, spleen, lungs, kidneys) and infected tissues were excised for ex vivo imaging to evaluate probe distribution and metabolism.

3.10. Antibacterial Activity of **Dman-C11 NPs** in vivo

Evaluation of **Dman-C11 NPs** Mediated Photodynamic Therapy against Skin Infections *i*n Vivo

The ethics committee approved the studies, which were conducted in accordance with the guidelines from the Institute of Materia Medica, Chinese Academy of Medical Sciences and Peking Union Medical College (approval number: 00003784). Male BALB/c mice, aged 6 to 8 weeks and weighing approximately 20 g, were purchased from Huafukang Biotechnology Co. Limited Company (Beijing, China). Full-thickness skin excision wounds, approximately 0.8 cm^2 in size, were created on the backs of each mouse. Subsequently, 100 μL of the MRSA bacterial suspension ($1×10^8$ CFU) was injected into the wounds. After 24 hours post intervention, treatments were performed on days 1, 3 and 5 : group 1 received 100 μL of PBS; group 2 was treated with a laser after receiving 50 μL of PBS (λ_{ex} = 655nm, 200 mW/cm^2, 10 min); group 3 received an injection of 100 μL of **Dman-C11 NPs** (10 μM); and group 4 underwent laser treatment after receiving 100 μL of **Dman-C11 NPs** (10 μM; the same laser irradiation as group 2). A portable camera was used to take daily photos of the wounds and surrounding area. Skin samples were collected from wound sites for immunofluorescence staining of TNF-α and IL-1β, as well as hematoxylin-eosin (H&E), Masson's trichrome staining and CD31immunohistochemical staining. Skin tissues from each group were weighed and homogenized in PBS buffer. The resulting suspensions were plated on LB agar plates and observed for bacterial growth after 24 h.

Evaluation of **Dman-C11 NPs** Mediated Photodynamic Therapy against Corneal Infections in Vivo

The ethics committee approved the studies, which were conducted in accordance with the guidelines from the Institute of Materia Medica, Chinese Academy of Medical Sciences, and Peking Union Medical College (Beijing; Approval Number: 00006355). Male C57 mice, aged 6 to 8 weeks, were purchased from Huafukang Biotechnology Co. Limited Company (Beijing, China). Before the surgery, C57 mice were anesthetized using isoflurane. The C57 mice MRSA-infected refractory keratitis animal model was established by injecting 10 μL of MRSA suspension (10^5 CFU/mL) into a mechanically injured C57 mice cornea. The C57 mice with keratitis were randomly divided into five groups (four animals per group): control, PBS, PBS+Laser, **Dman-C11**, **Dman-C11**+Laser and Ofloxacin. The anterior segment was measured with a slit lamp at 1, 3, 5, and 7 days after treatment. Postoperative inflammation was clinically evaluated. After the final treatment, all C57 mice were euthanized, and their eyeballs were taken for a quantitative examination of the bacterial load. The homogenate was then serially diluted before being plated on LB agar. The number of colonies on the plate was counted after 12 h of incubation at 37 ºC to assess the bacteria load of each eye.

3.11 Flow Cytometry Analysis

RAW 264.7 cells were seeded in 6-well plates at a density of $1 × 10^5$ cells per well and cultured overnight. The medium was then replaced with fresh DMEM, and the cells were treated under different conditions (e.g., IL-4, high/low MRSA stimulation) for 2 h. After washing three times with PBS, cells were blocked with an anti-CD16/32 antibody (Solarbio, 1 μL per 100 μL for ~$1 × 10^{-6}$ cells). Cells were then fixed and permeabilized using the Cyto-Fast Fix/Perm Buffer Set (BioLegend), followed by staining on ice with a PE-conjugated anti-CCR7 antibody (2 μL per 100 μL for ~$1 × 10^{-6}$ cells, incubated for 10 min) and an APC-conjugated anti-CD206 antibody (Elabscience, 5 μL per 100 μL for ~$1 × 10^{-6}$ cells) for 30 min. After staining, cells were washed

and analyzed by flow cytometry. APC was excited by a red laser (627–640 nm) and detected using an optical filter centered at 660 nm (e.g., 660/20 nm bandpass filter). Data were analyzed using IDEAS 6.0 software.

Results and Discussion

Table S1. Minimum inhibitory concentrations (MIC) of **Dman-C11 NPs**.

MIC/μM	*S. aureus*	MRSA
Dman-C11	>25	>25

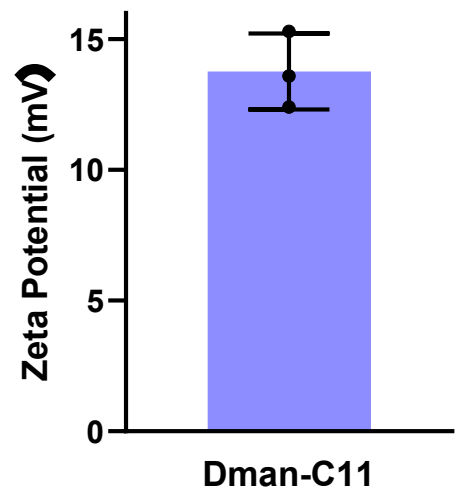

Figure S1. Zeta potential of **Dman-C11** in deionized water.

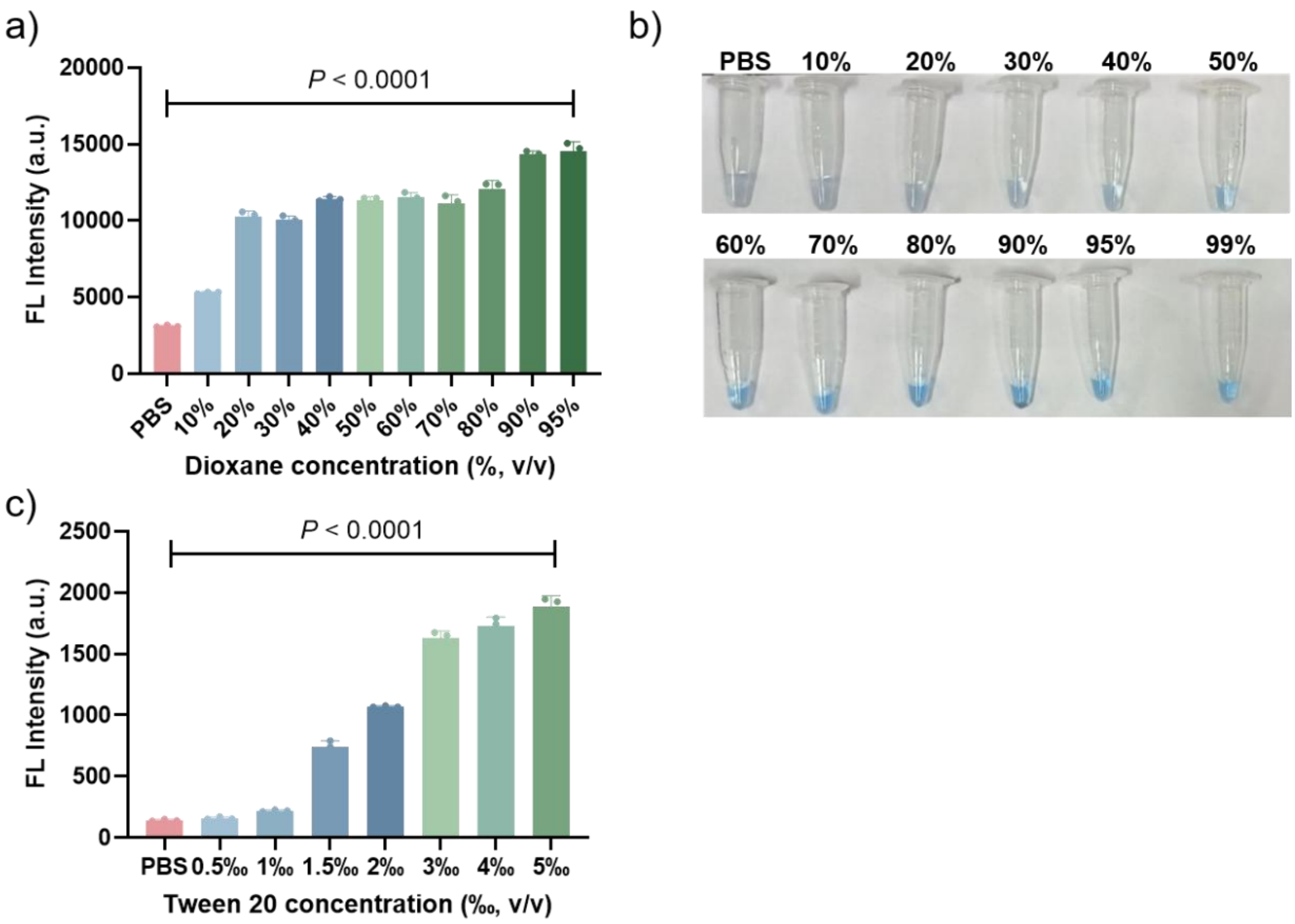

Figure S2. Effects of 1,4-dioxane and Tween 20 on the disassembly of **Dman-C11 NPs**. (a) Fluorescence intensities of **Dman-C11** in PBS containing increasing concentrations of 1,4-dioxane (10–95%, v/v). (b) Corresponding photographs of the solutions under ambient light. (c) Fluorescence intensities of **Dman-C11** in PBS containing increasing concentrations of Tween 20 (0.5–5‰, v/v). Data are presented as the mean ± SD (n = 3). Statistical significance was determined using one-way ANOVA followed by Tukey's multiple-comparison test; exact P values are indicated in the figure.

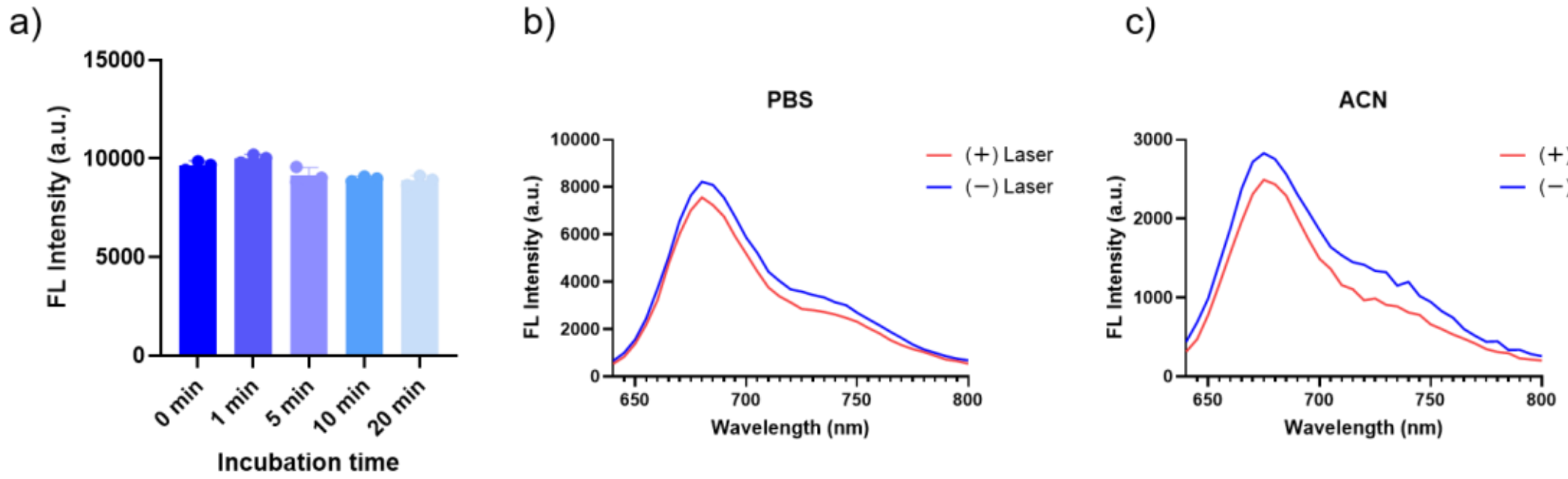

Figure S3. Probe stability. (a) Fluorescence intensity of the probe after incubation with MRSA for different periods (0, 1, 5, 10, and 20 min). After incubation, the precipitates were collected by centrifugation, dissolved in a methanol/acetonitrile (1:1, v/v) mixture, and **analysed** by fluorescence emission spectroscopy. (b, c) Fluorescence emission spectra of the probe (10 μM) in PBS (b) and ACN (c) before (blue) and after (red) irradiation at 200 mW cm^{-2} for 7 min.

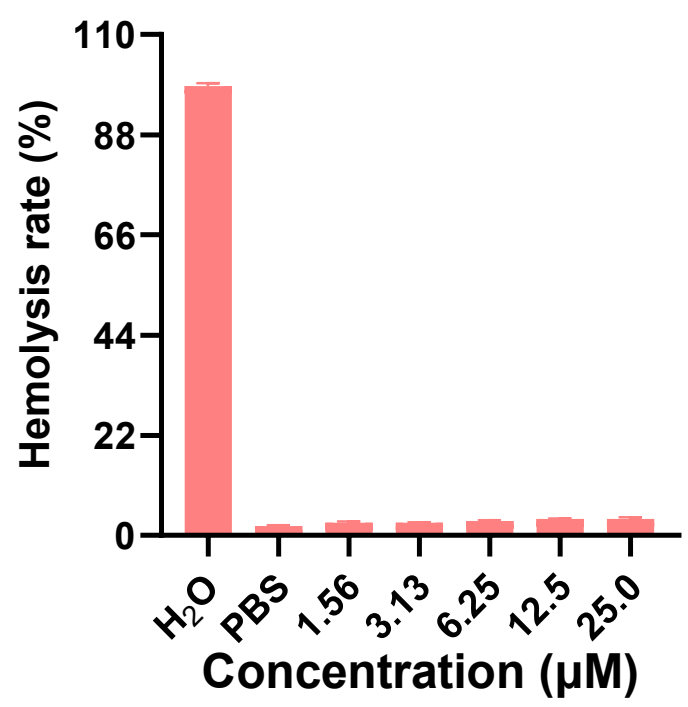

Figure S4. Hemolysis rates of **Dman-C11 NPs** at different concentrations. ddH_2O and PBS were used as a positive control and negative control, respectively.

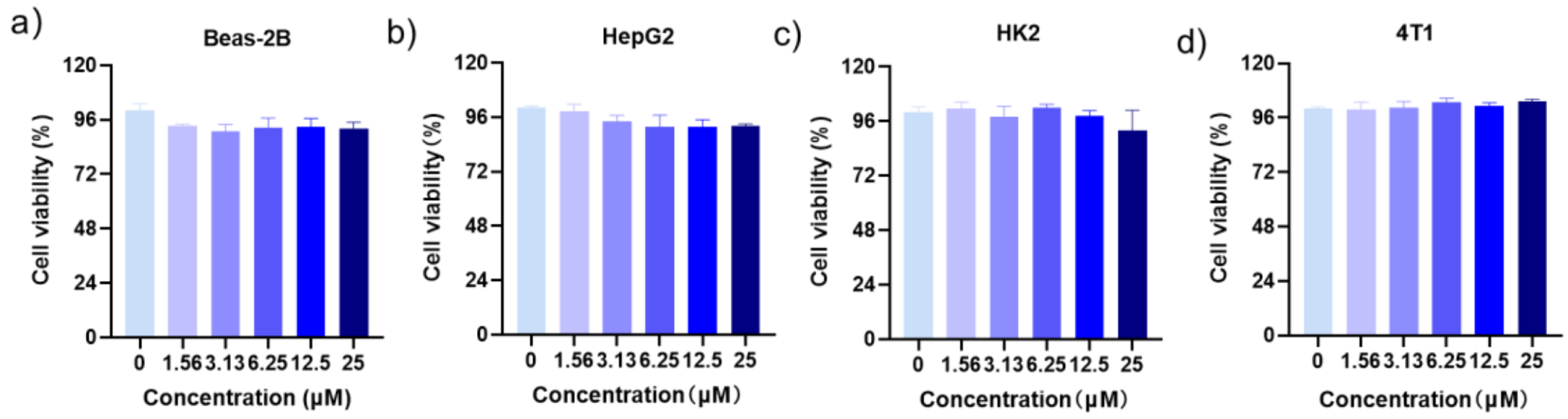

Figure S5. Cell viability of different cells incubated with **Dman-C11 NPs** at different concentrations.

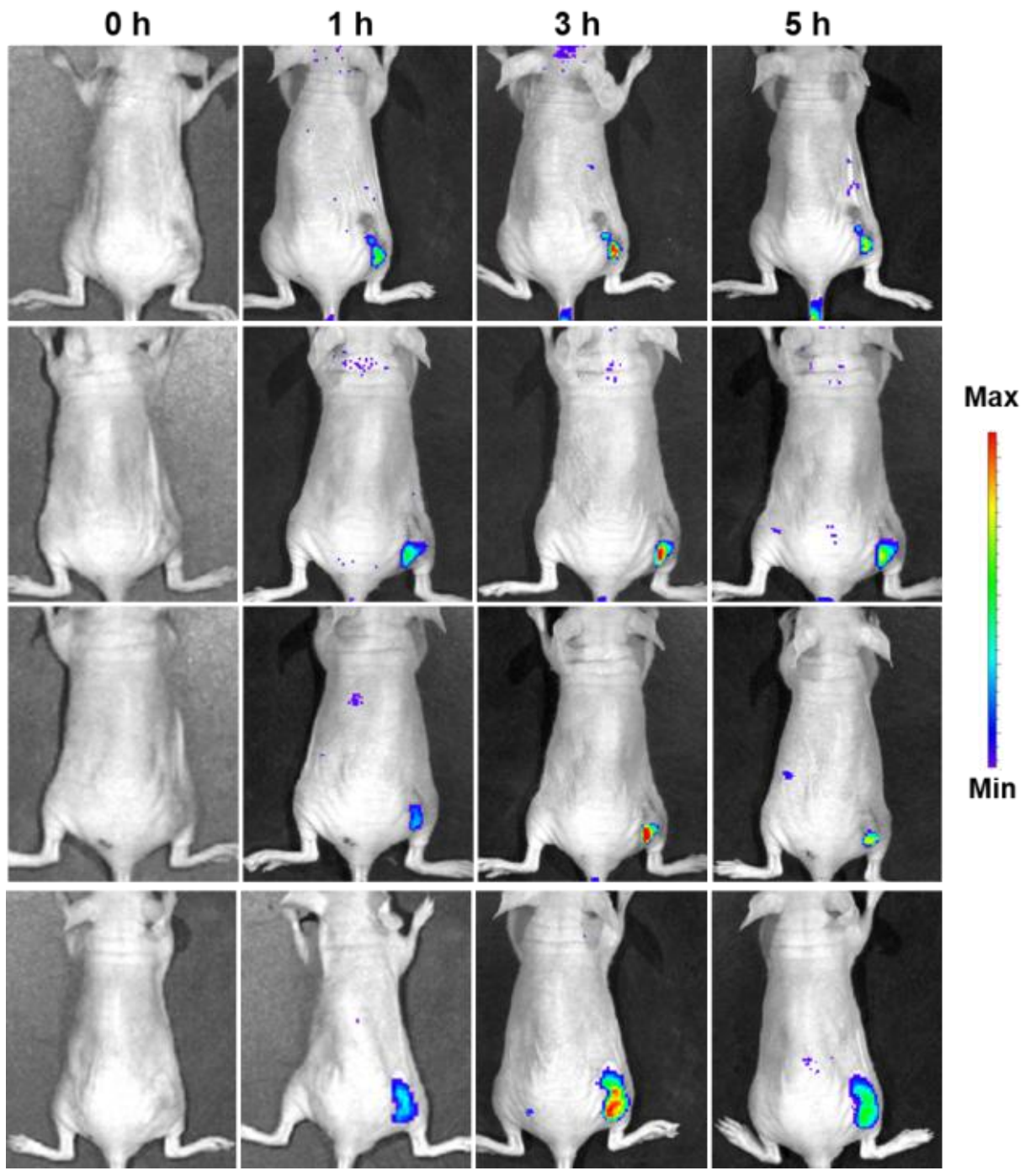

Figure S6. NIR fluorescence images of the mice upon subcutaneous injection of **Dman-C11 NPs** (data represent mean ± SD, n = 4 independent mice).

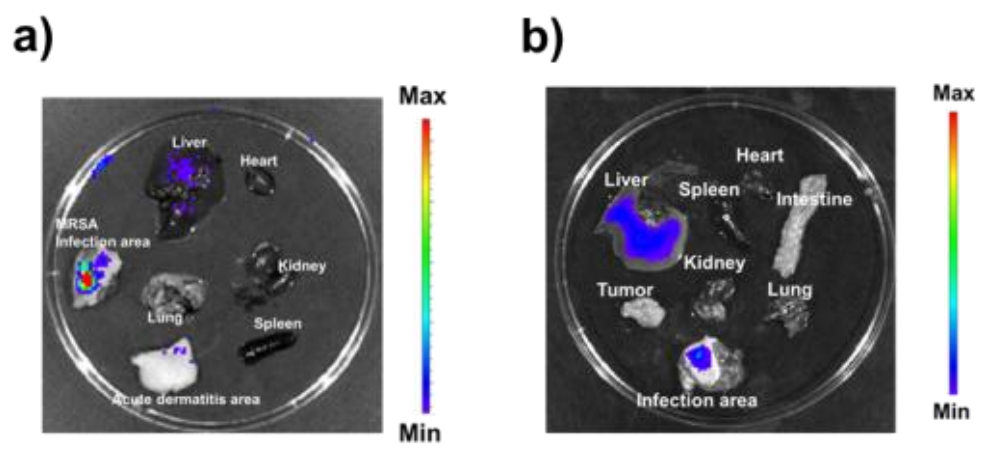

Figure S7. Fluorescence intensity of different parts in a) acute dermatitis model and b) 4T1 breast cancer model.

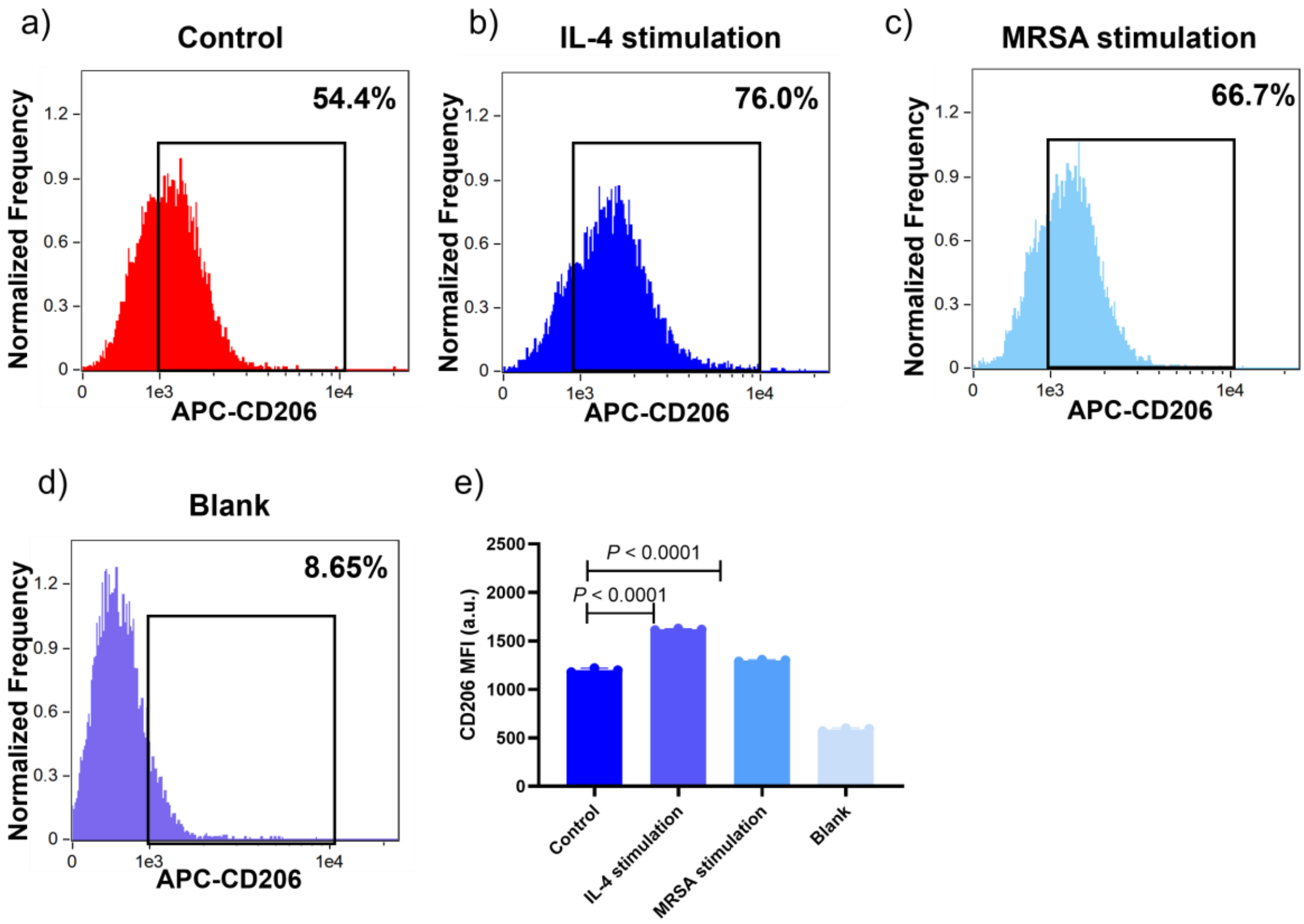

Figure S8. Flow-cytometric analysis of CD206 expression in RAW264.7 macrophages. (a–d) Representative histograms of APC-conjugated anti-CD206 staining in the Control, IL-4, MRSA (1.0×10^7 CFU mL^{-1}), and unstained Blank groups, respectively. The boxed regions and numbers indicate the percentages of CD206-positive cells. (e) Quantification of CD206 mean fluorescence intensity (MFI). Data are presented as the mean ± SD (n = 3). Statistical significance was determined by one-way ANOVA followed by Dunnett's multiple-comparisons test versus the Control group; P values are indicated in the figure.

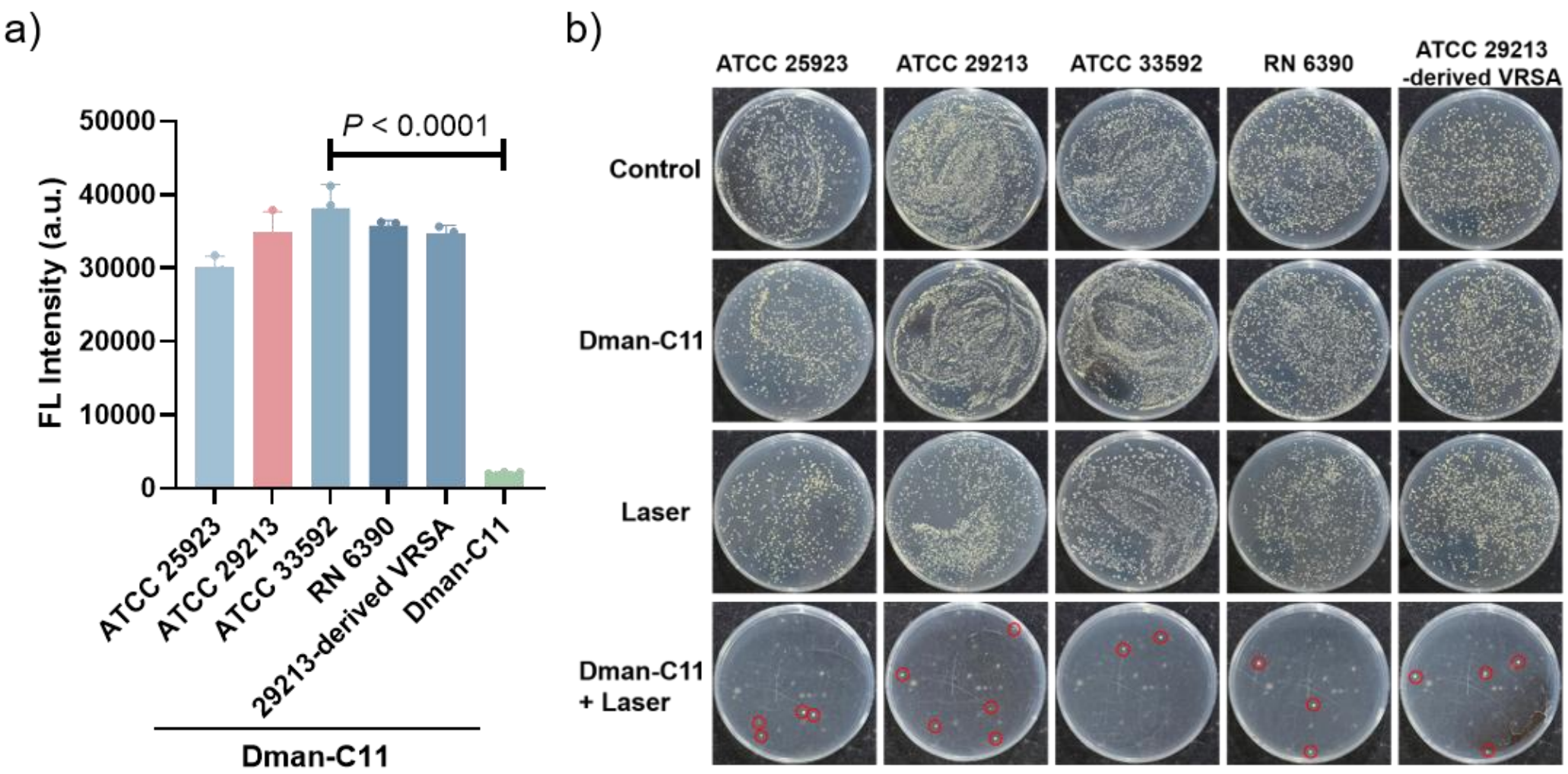

Figure S9. Fluorescence response of **Dman-C11** to different *S. aureus* strains and in vitro photodynamic antibacterial activity of **Dman-C11 NPs**. (a) Fluorescence intensity of **Dman-C11**

NPs after incubation with different *S. aureus* strains: ATCC 25923, RN 6390, ATCC 29213, ATCC 33592, and a laboratory-induced VRSA derivative of ATCC 29213. **Dman-C11 NPs** without bacteria served as the probe-only control. Data are presented as the mean ± SD (n = 3). Statistical significance was determined using one-way ANOVA followed by Tukey's multiple-comparison test; $P < 0.0001$ for ATCC 33592 versus the probe-only control. (b) Representative images of bacterial colonies on agar plates after treatment of the five *S. aureus* strains with PBS (control), **Dman-C11 NPs**, laser irradiation alone, or **Dman-C11 NPs** plus laser irradiation. Red circles indicate bacterial colonies in the **Dman-C11 NPs** plus laser irradiation group. All plates were photographed on the same benchtop; surface wear and marks visible through the transparent Petri dishes are background features rather than bacterial colonies.

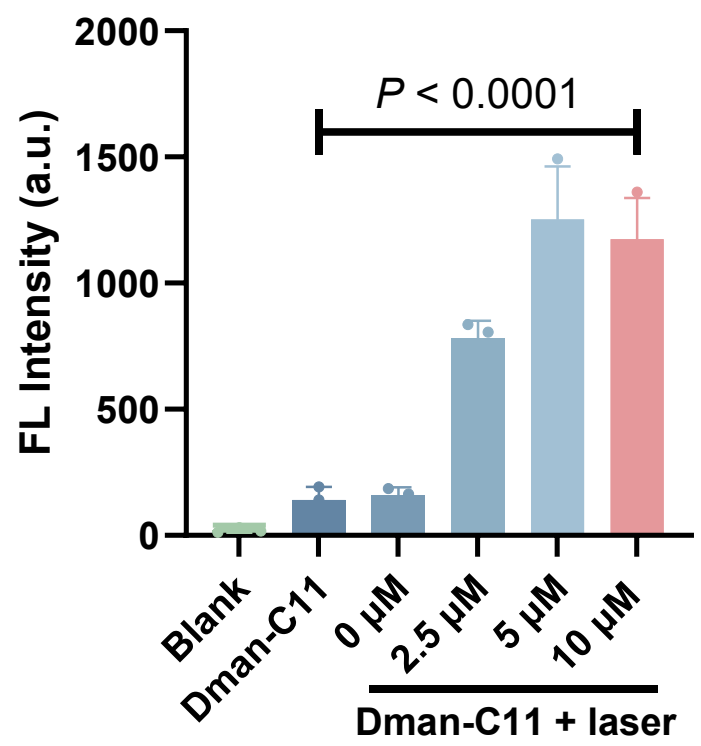

Figure S10. Cytoplasmic membrane depolarization induced by photoactivated **Dman-C11**. DiBAC4(3) fluorescence intensity in MRSA after different treatments. MRSA incubated with DiBAC4(3) only served as the blank control. and MRSA treated with **Dman-C11** (10 μM) without irradiation served as negative controls. The **Dman-C11** + laser groups were treated with different concentrations of **Dman-C11** (2.5, 5, and 10 μM) under NIR irradiation. The 0 μM group (irradiation-only control) received laser treatment without **Dman-C11**. Data are presented as the mean ± SD (n = 3). Statistical significance was determined using one-way ANOVA followed by Tukey's multiple-comparison test; exact P values are indicated in the figure.

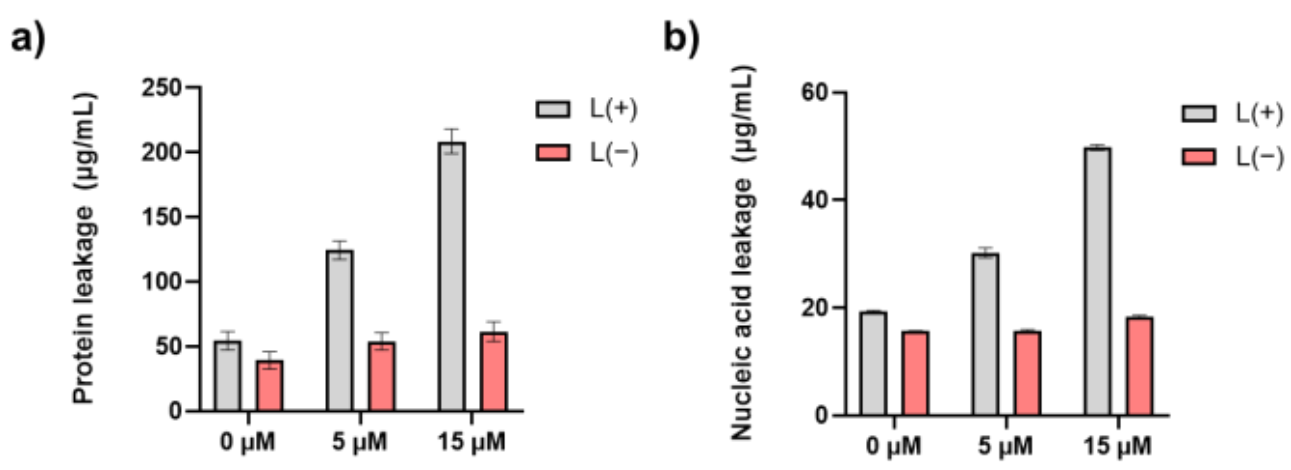

Figure S11. Protein leakage (a) and nucleic acid leakage (b) of *S. aureus* incubated with **Dman-C11 NPs** with or without NIR laser.

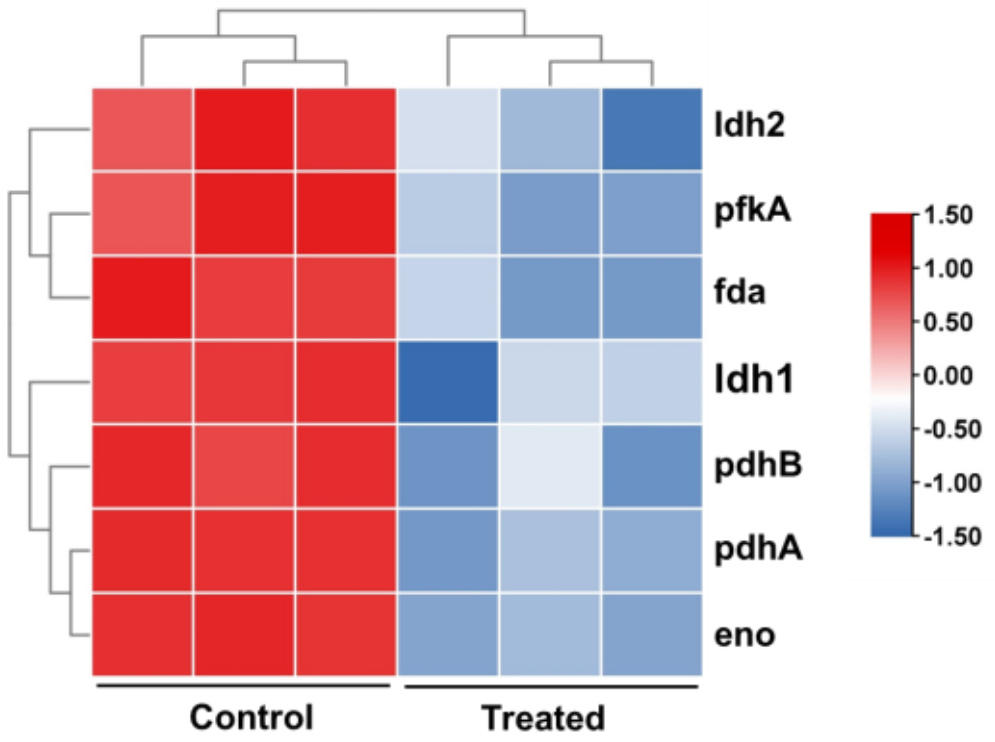

Figure S12. Heatmap of DEGs involved in glycolysis and pyruvate metabolism. The heatmap shows the expression profiles of representative DEGs related to glycolysis and pyruvate metabolism in MRSA after treatment. Red and blue indicate upregulated and downregulated genes, respectively.

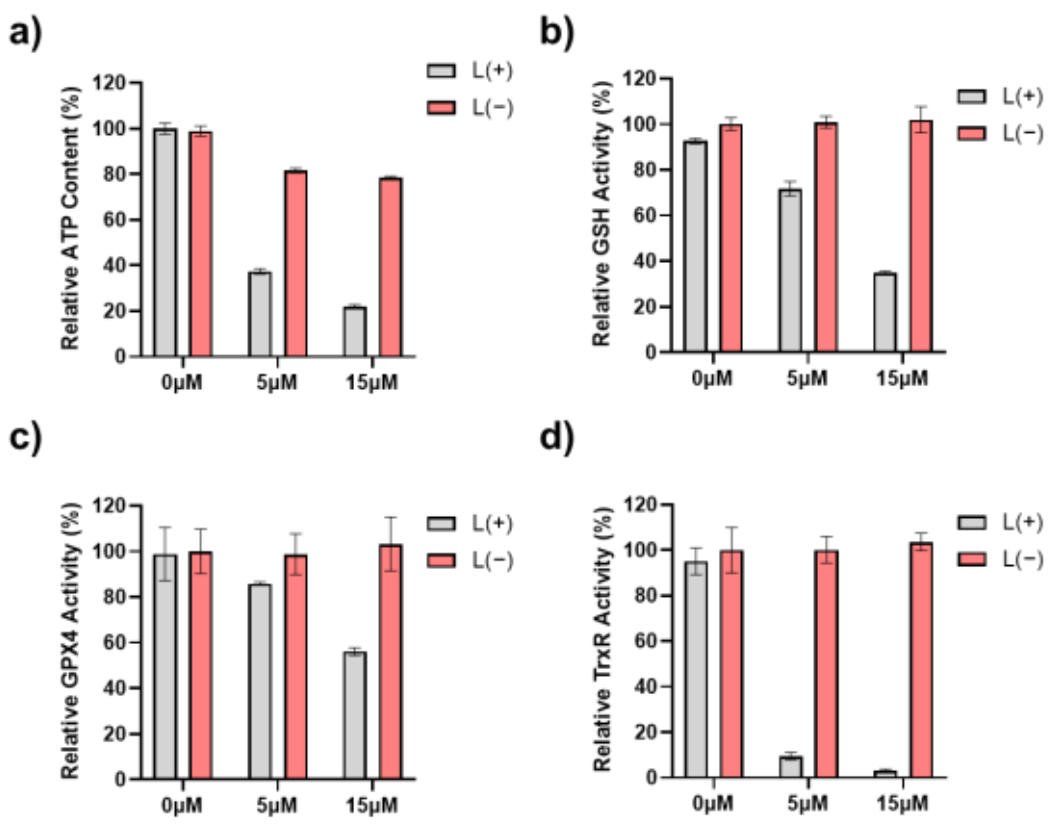

Figure S13. Intracellular ATP (a), GSH (b), GPX4(c) and TrxR (d) activity in *S. aureus* after **Dman-C11 NPs** treatment under NIR irradiation (L+) or non-irradiation (L−) at different concentrations.

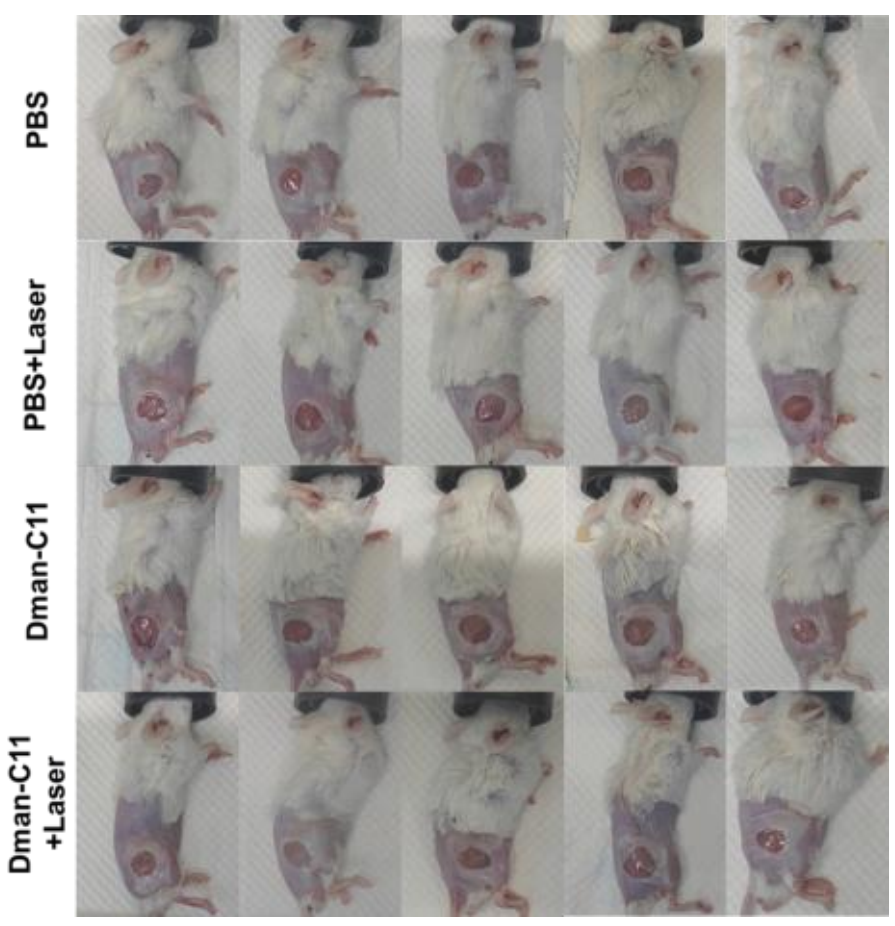

Figure S14. Photographs of the mice's skin wounds on the 1st day (data represent mean ± SD, n = 5 independent mice).

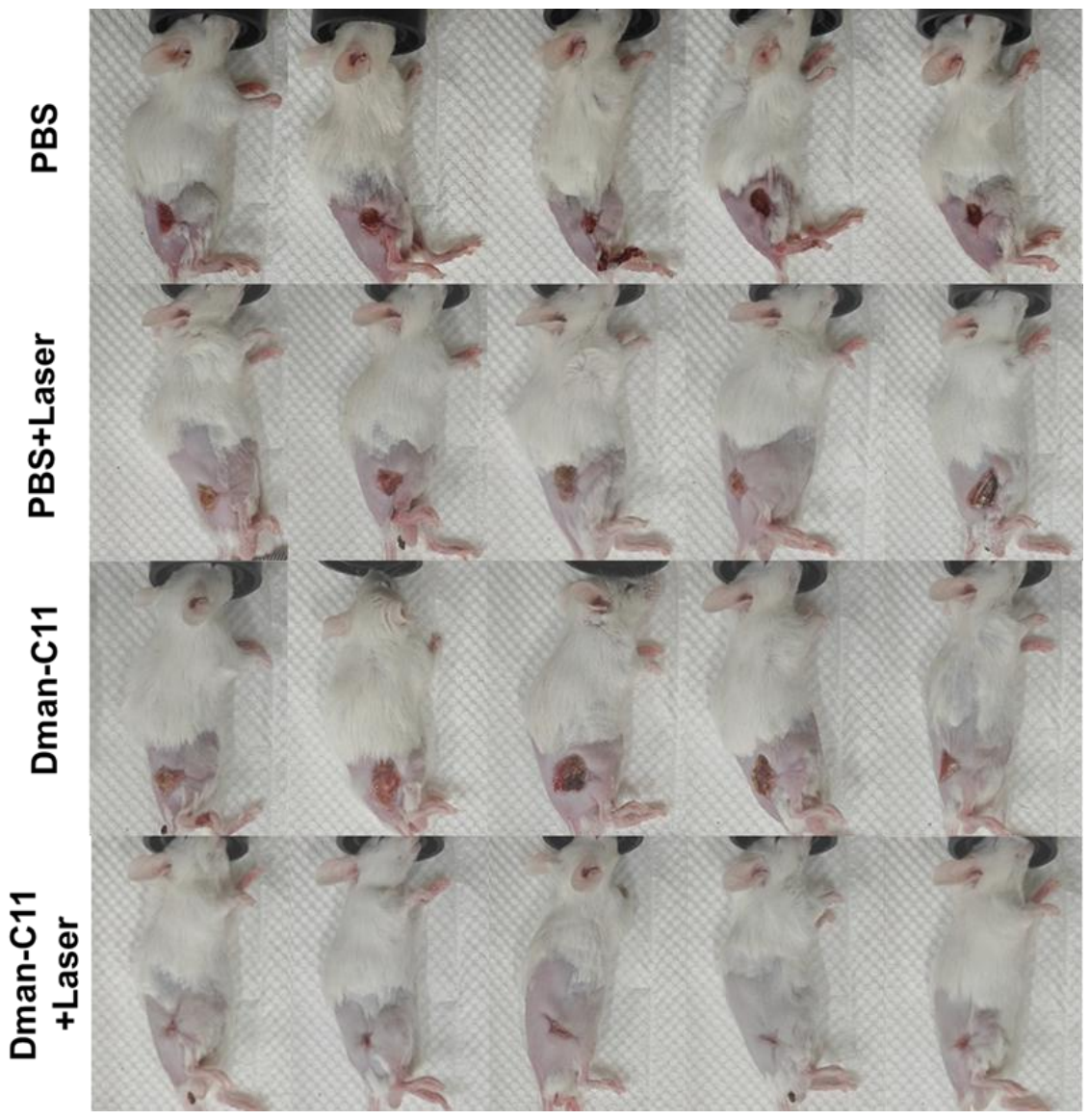

Figure S15. Photographs of the mice's skin wounds on the 13th day (data represent mean ± SD, n = 5 independent mice).

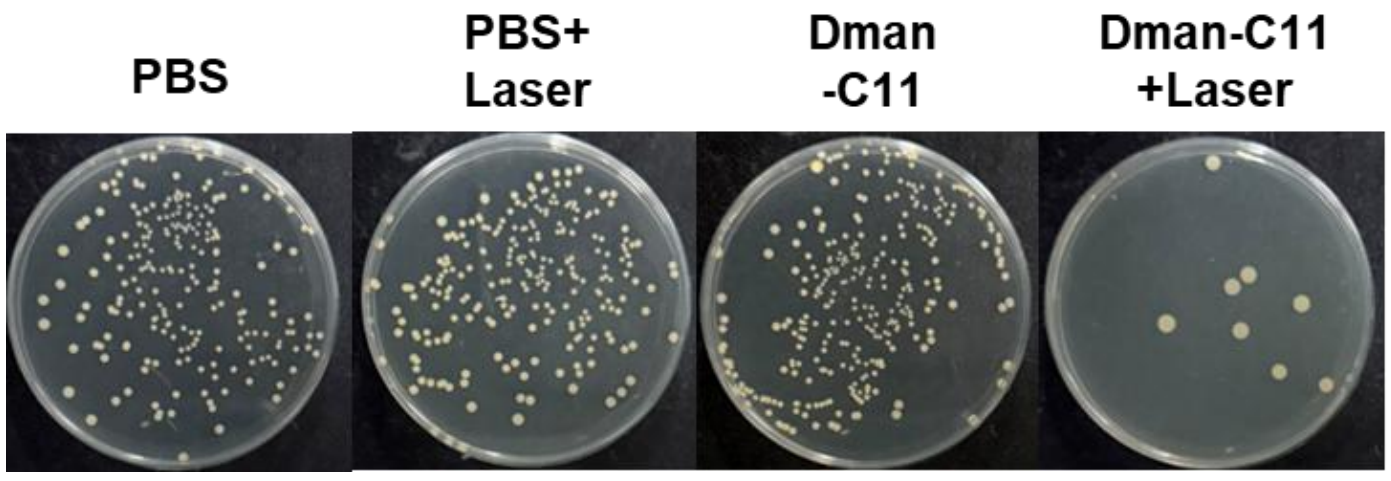

Figure S16. Colony formation photos of skin wounds on the 13th day.

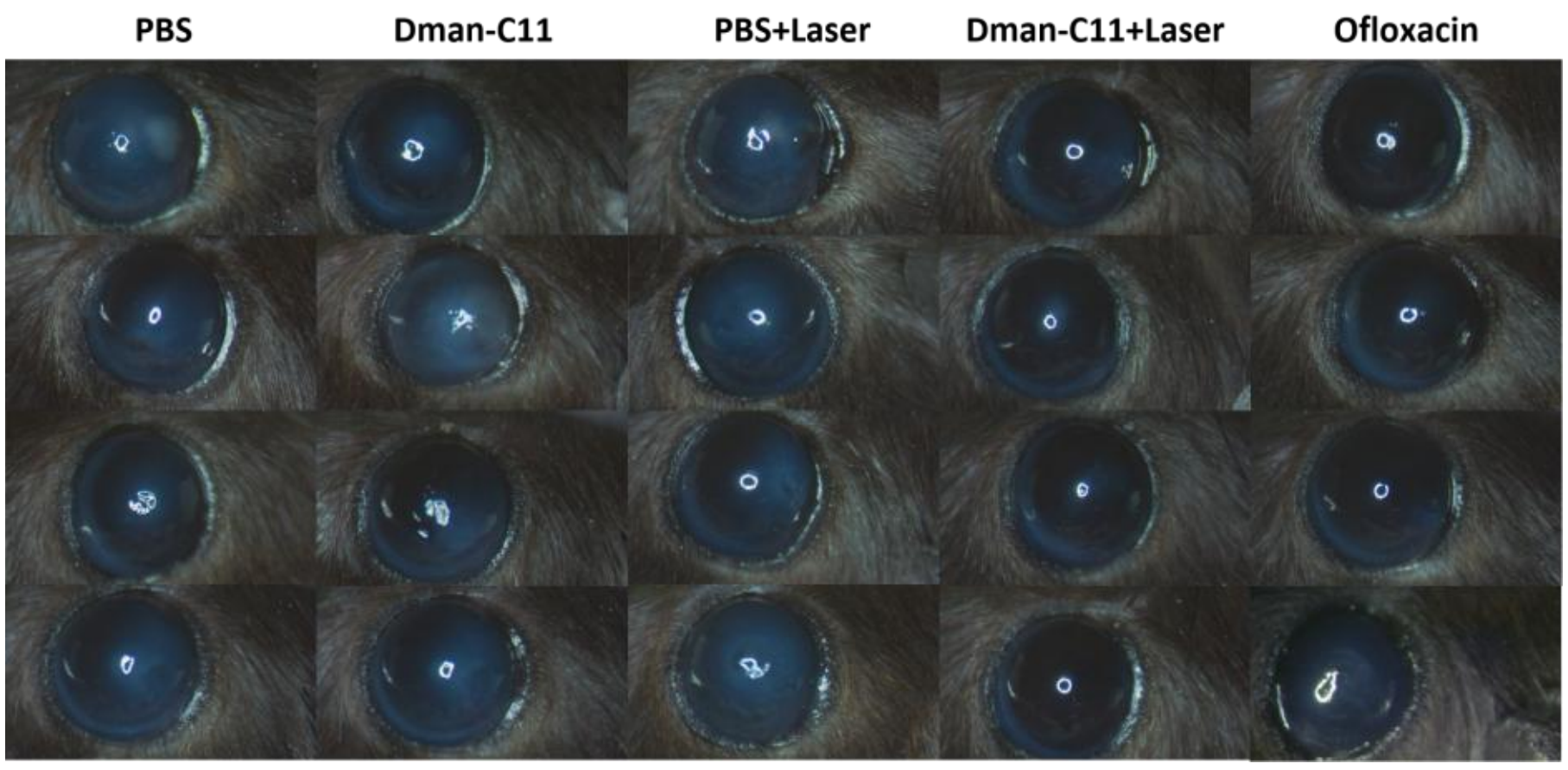

Figure S17. Photographs of the mice's corneal wounds on the 7th day (data represent mean ± SD, n = 4 independent mice).

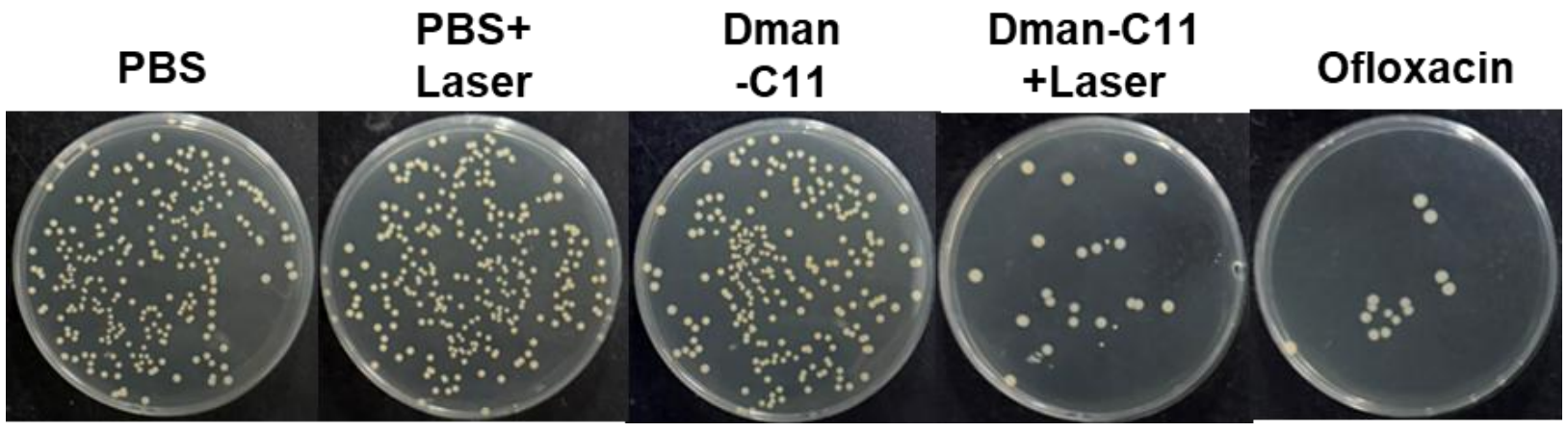

Figure S18. Colony formation photos of corneal wounds on the 7th day.

NMR and LC-MS Spectrum

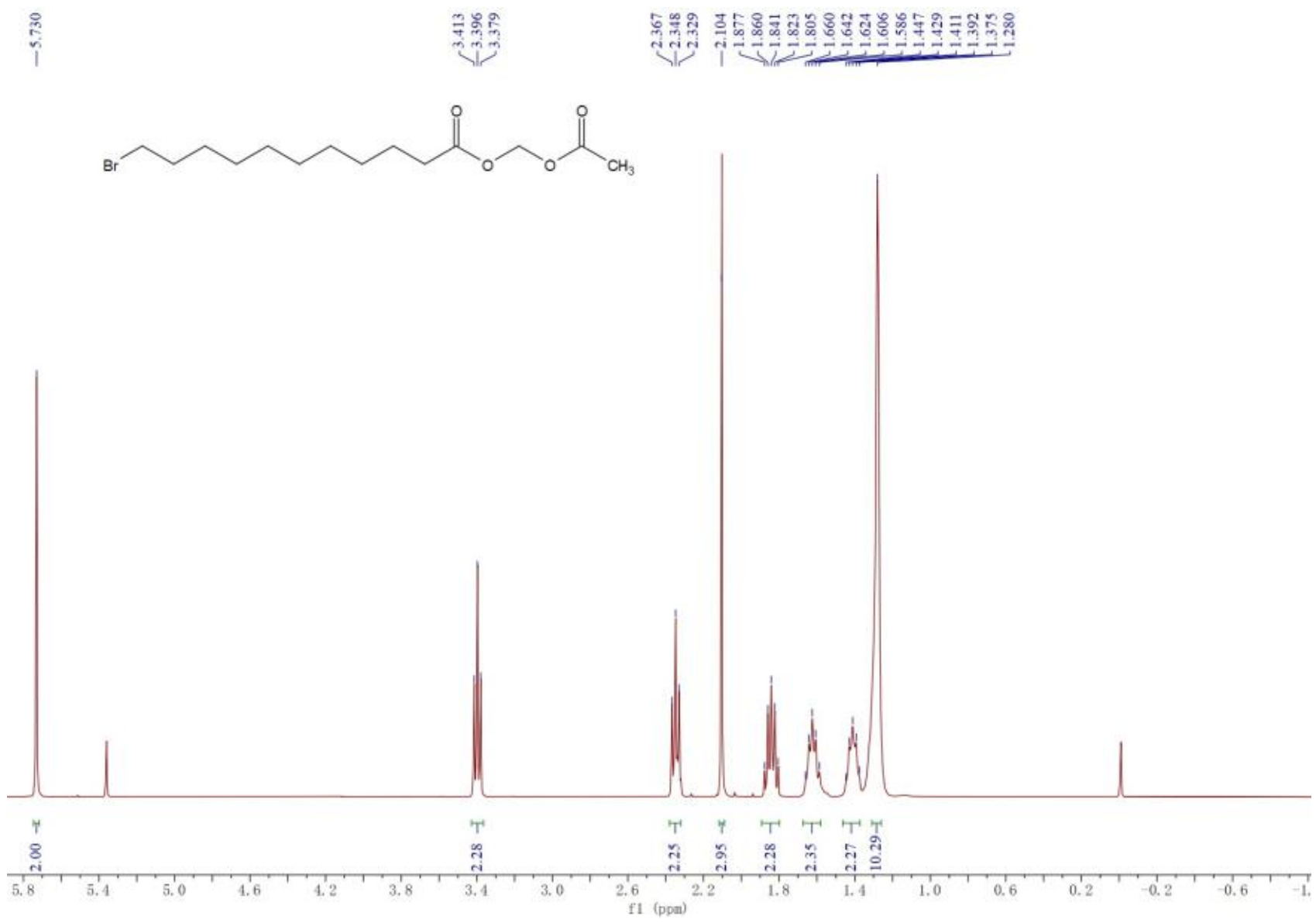

Figure S19. ^{1}H NMR spectrum of compound 1-3.

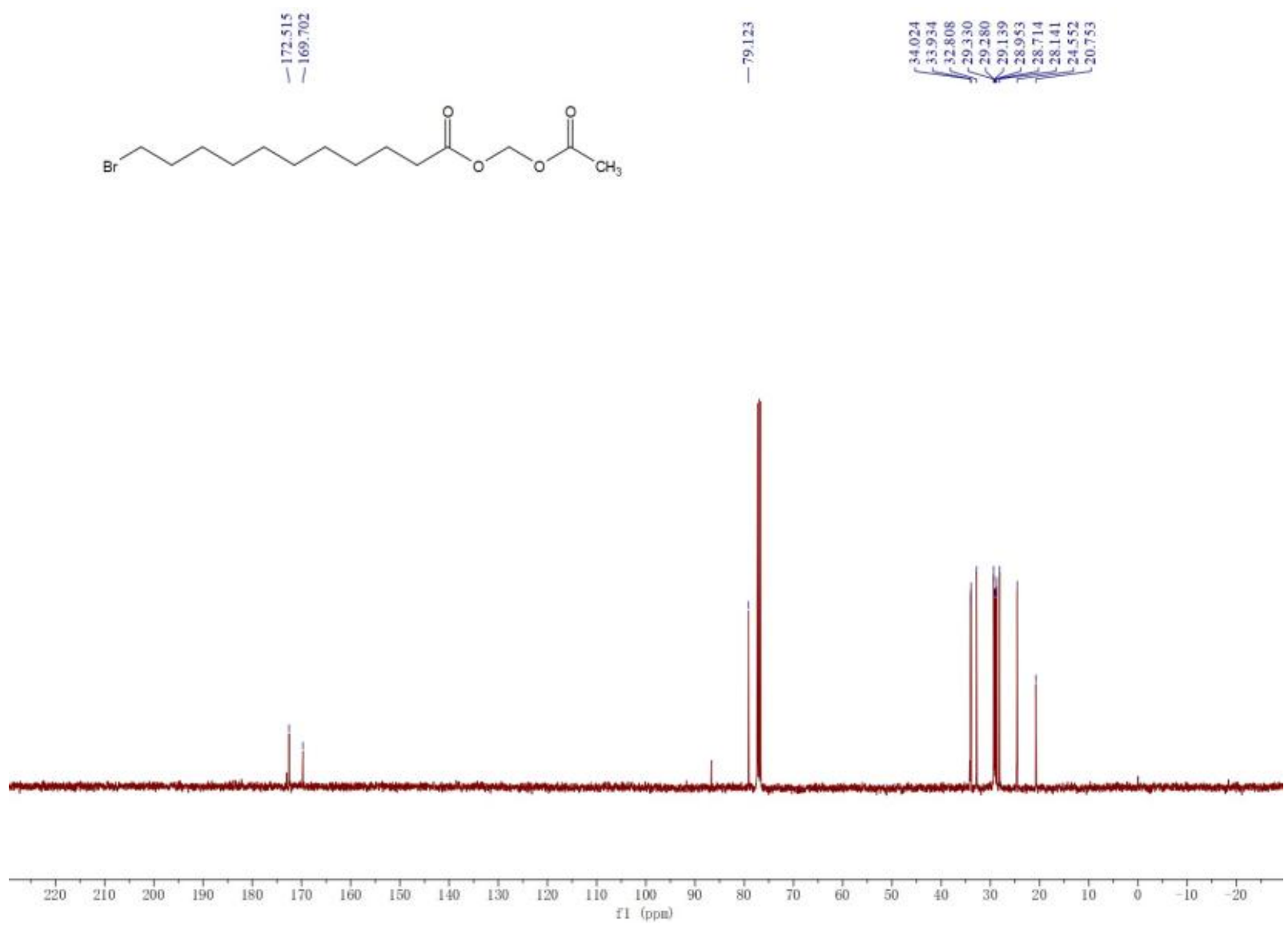

Figure S20. ^{13}C NMR spectrum of compound 1-3.

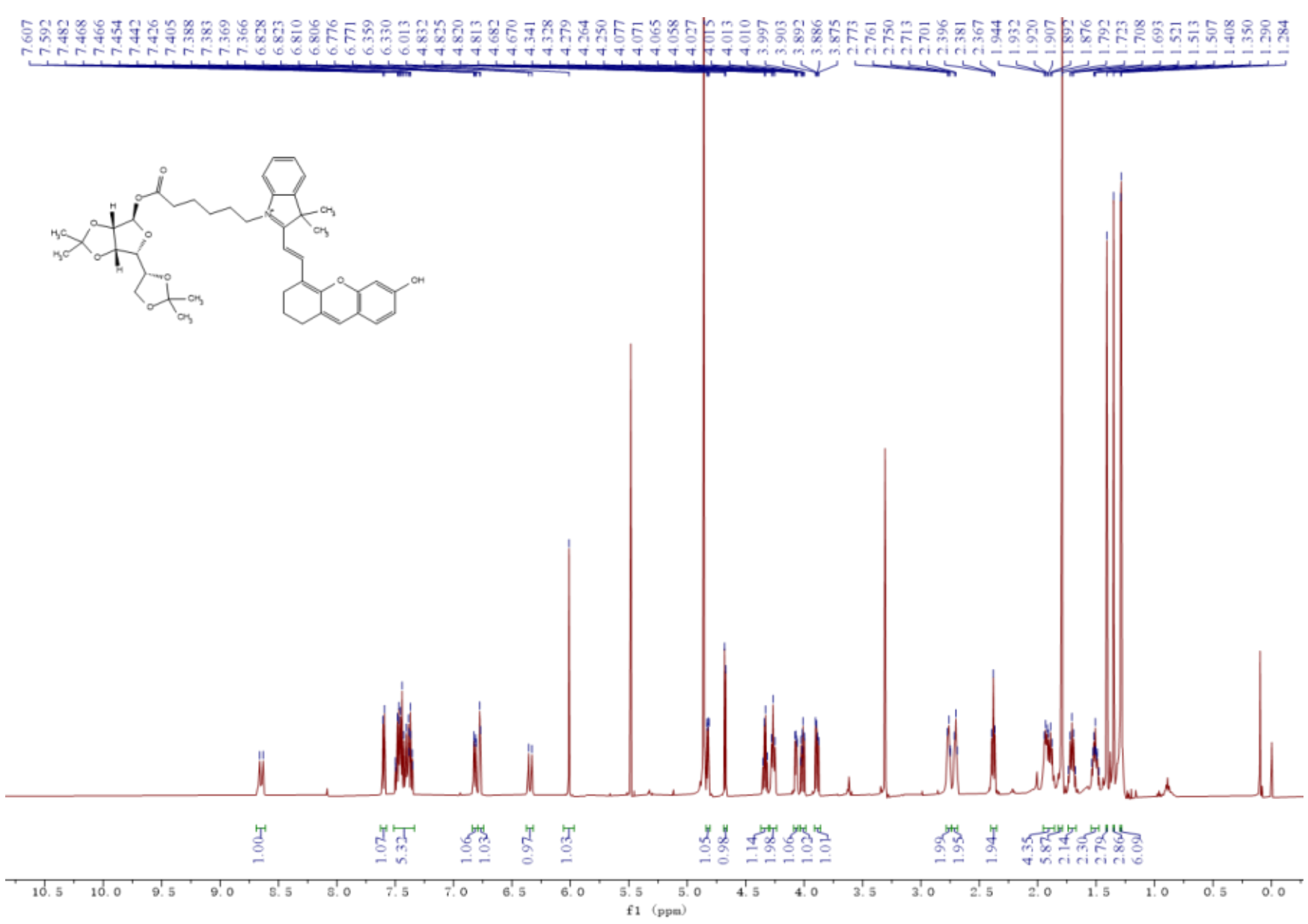

Figure S21. ^{1}H NMR spectrum of compound 1-6.

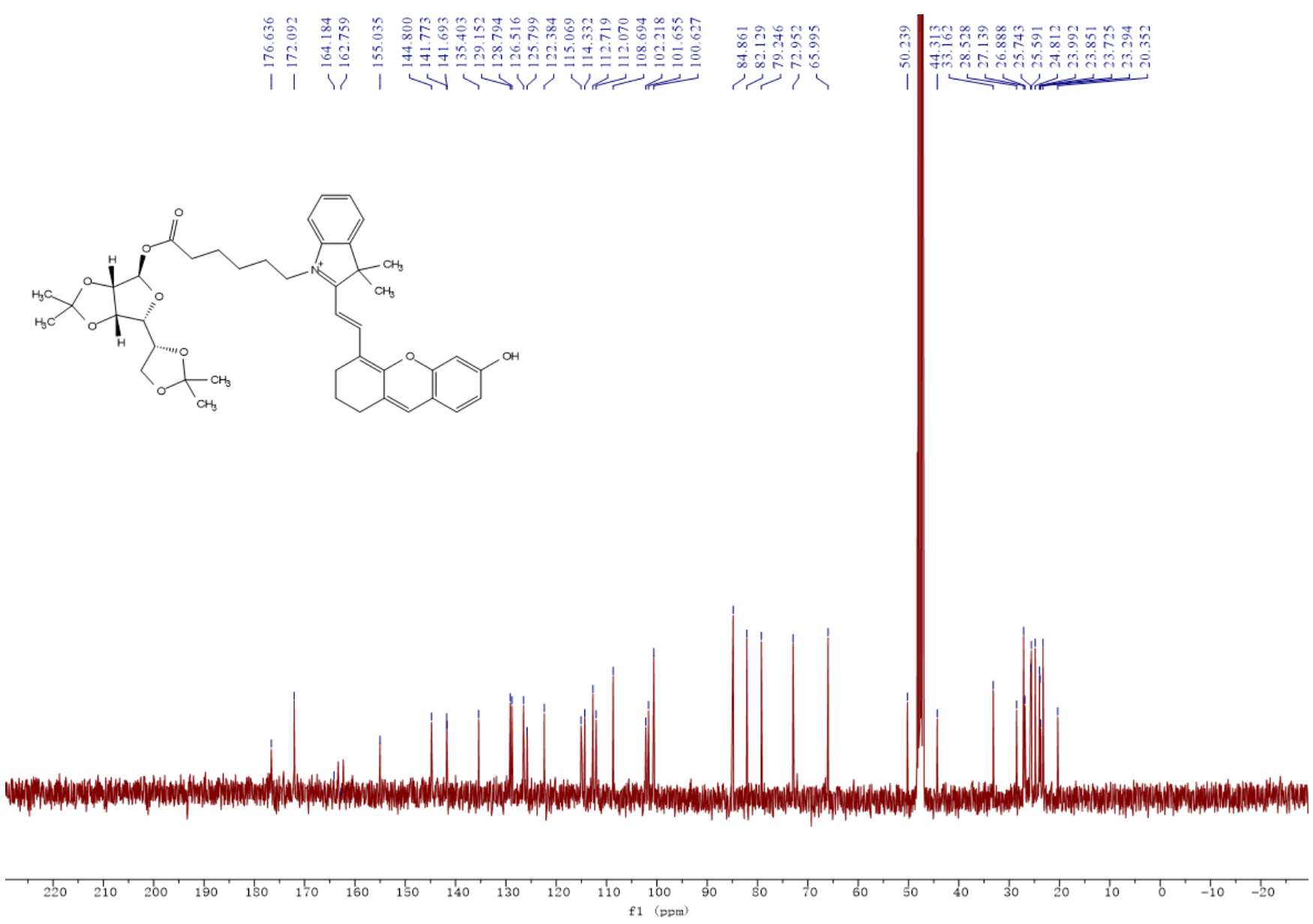

Figure S22. ^{13}C NMR spectrum of compound 1-6.

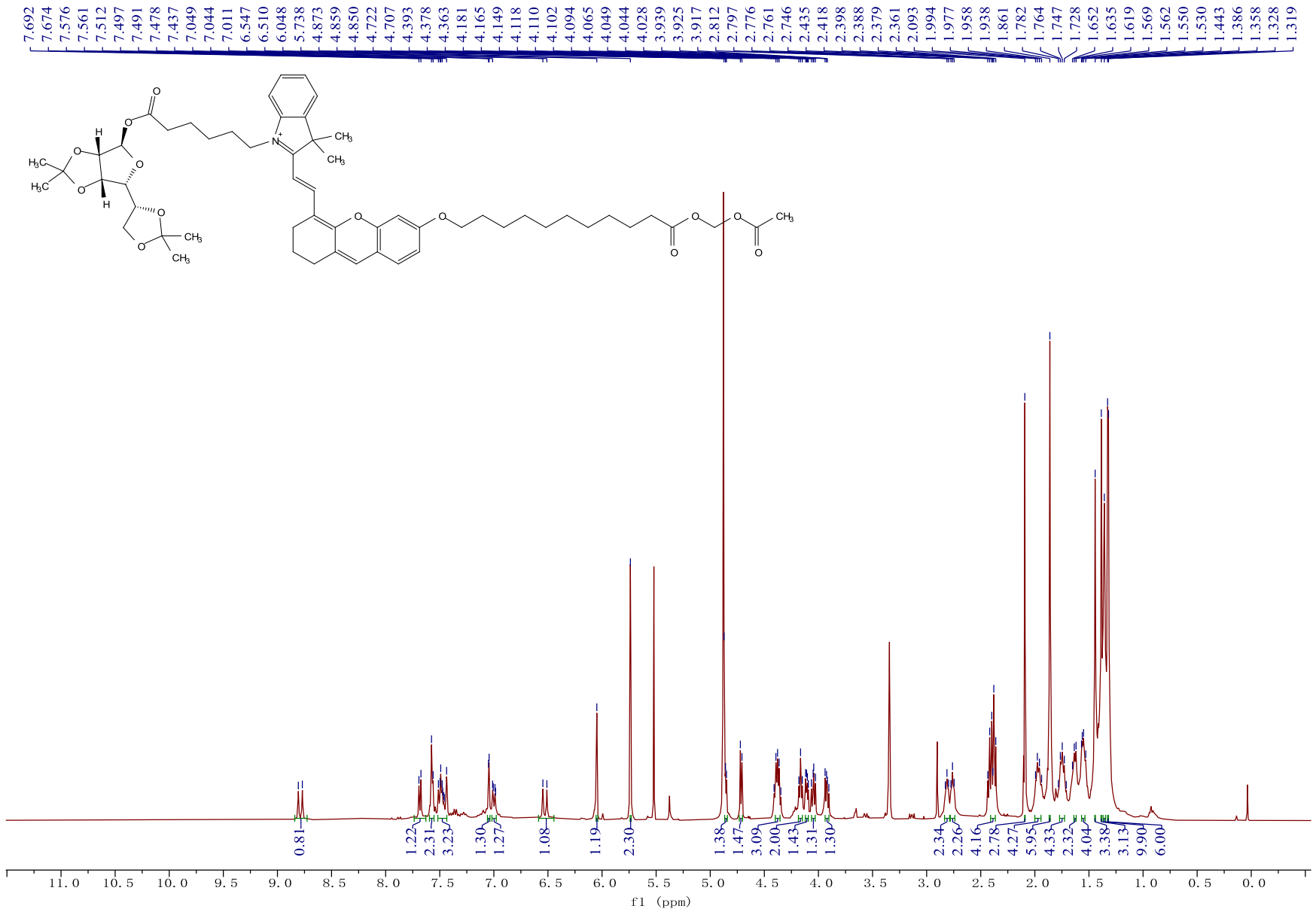

Figure S23. ^{1}H NMR spectrum of compound 1-7.

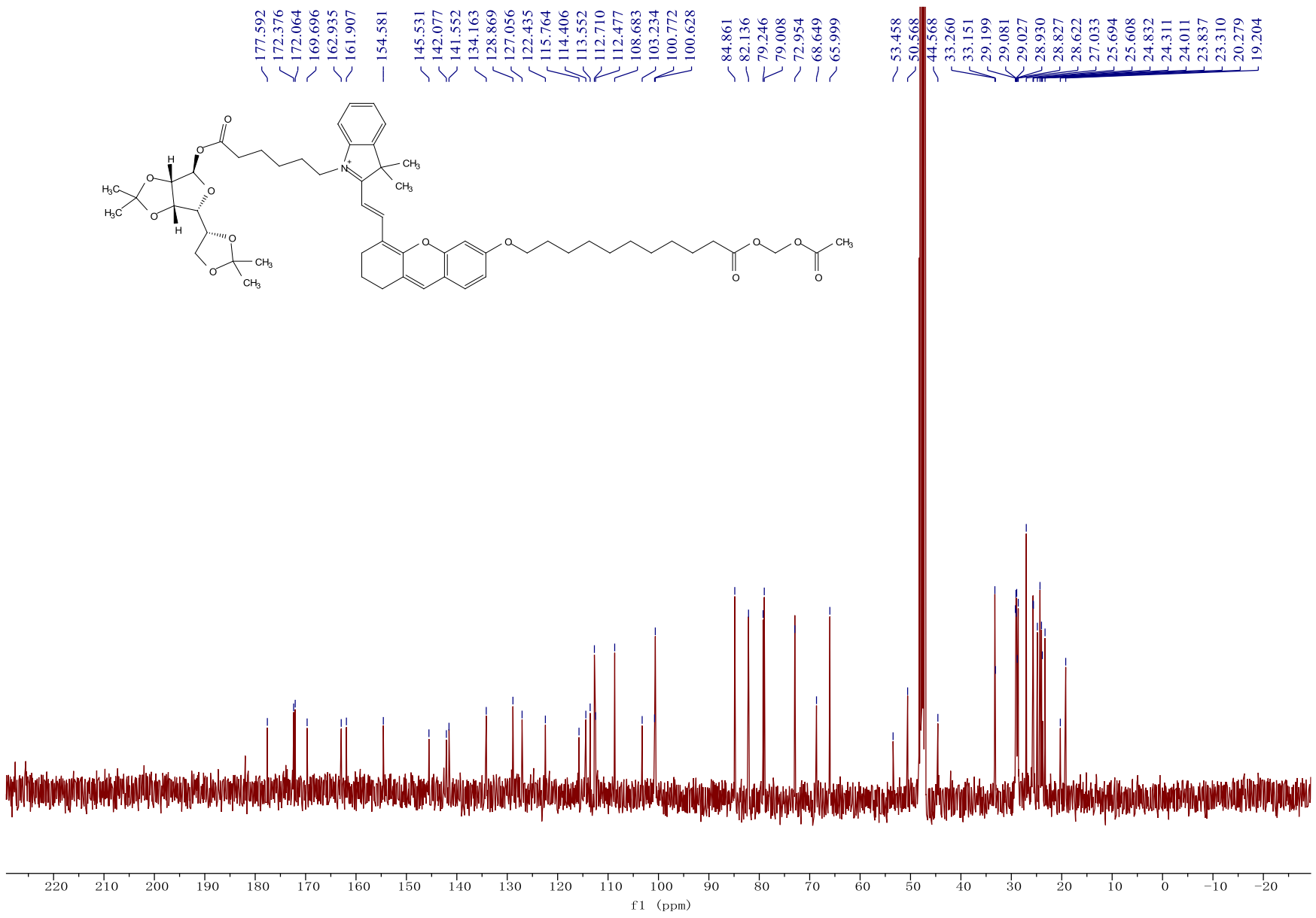

Figure S24. ^{13}C NMR spectrum of compound 1-7.

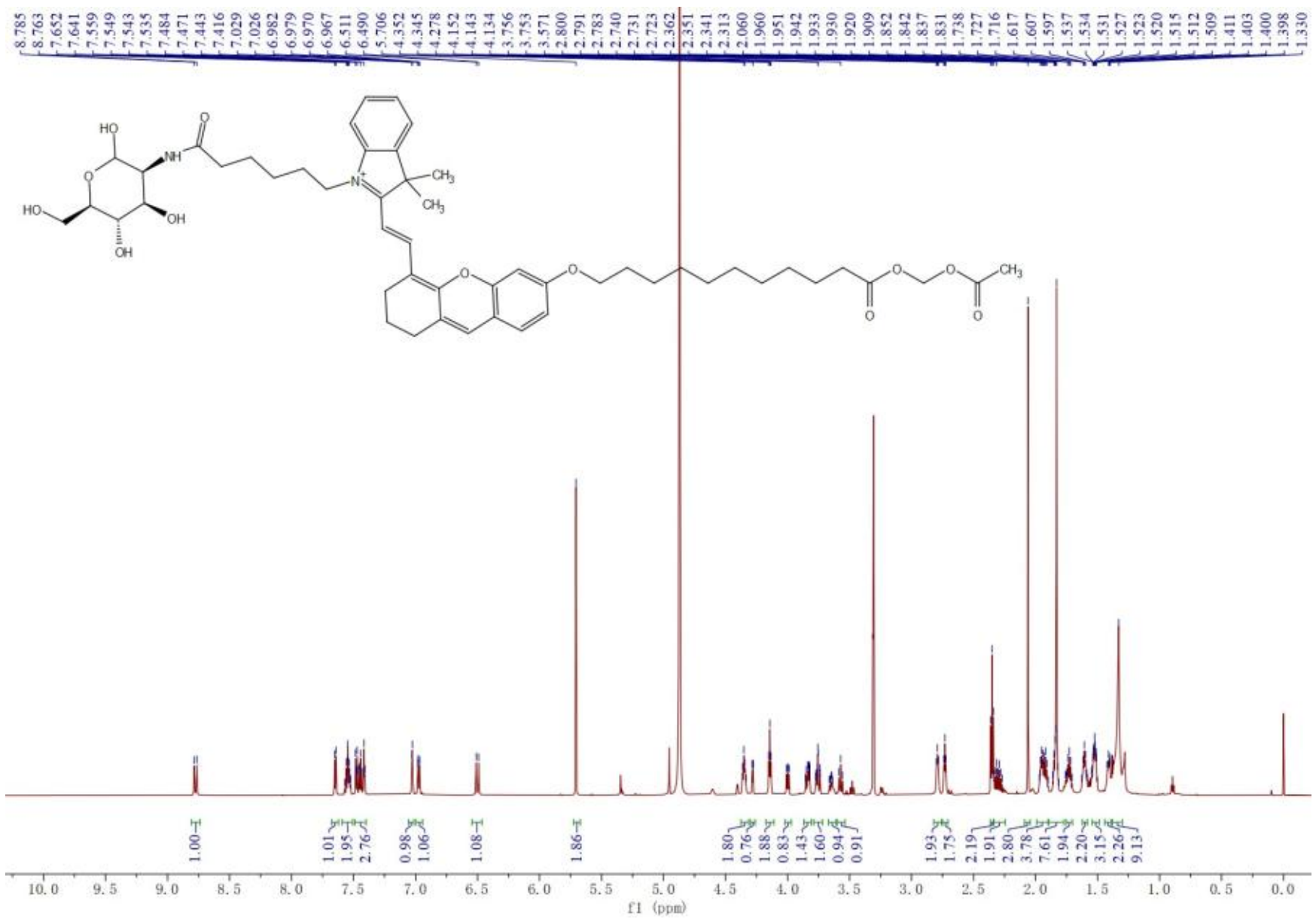

Figure S25. ^{1}H NMR spectrum of compound **Dman-C11**.

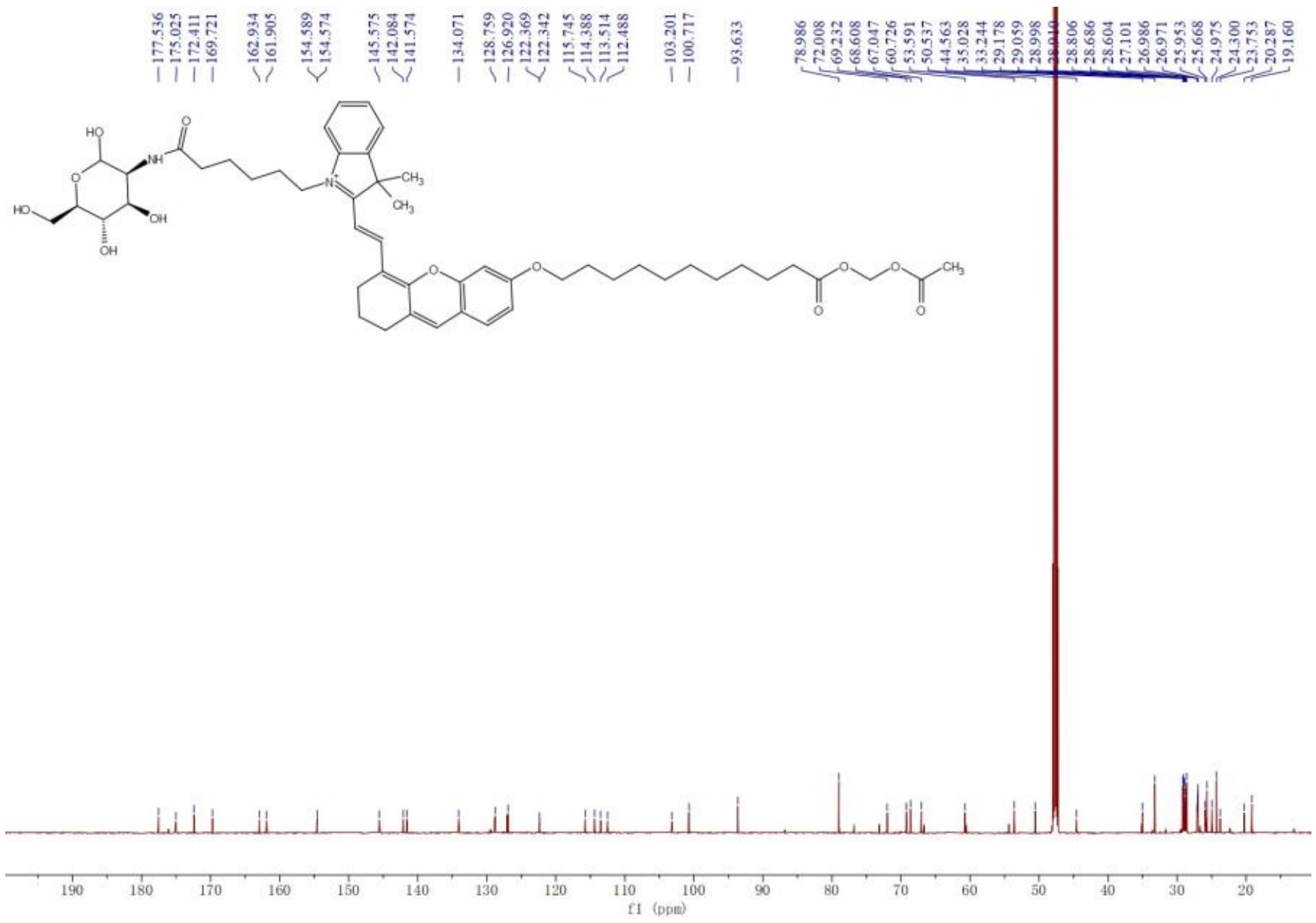

Figure S26. ^{13}C NMR spectrum of compound **Dman-C11**.

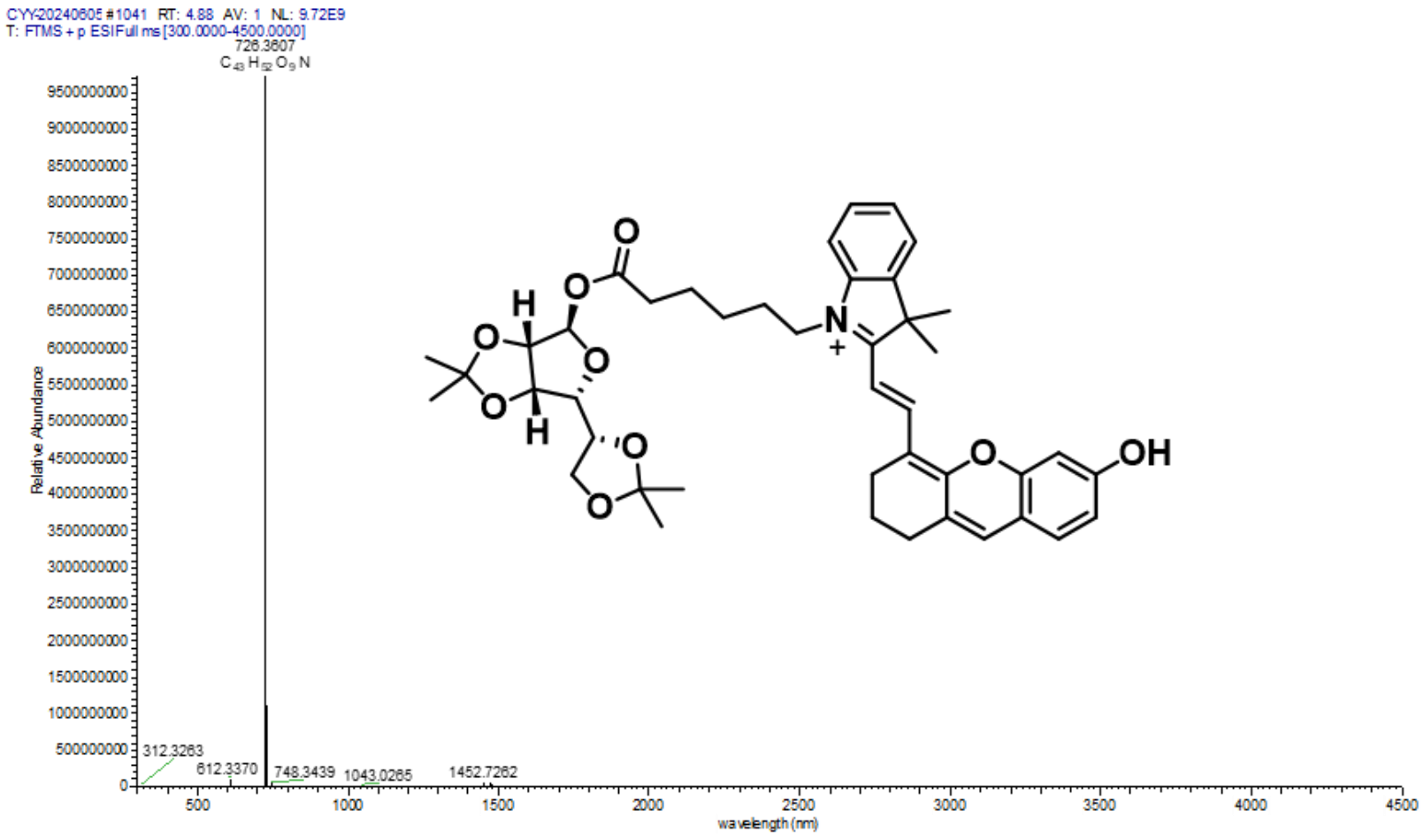

m/z	Theo. Mass	Delta (ppm)	RDB equiv.	Composition
726.3607	726.3637	-4.05	18.5	C43 H52 O9 N

Figure S27. LC-MS results of compound 1-6.

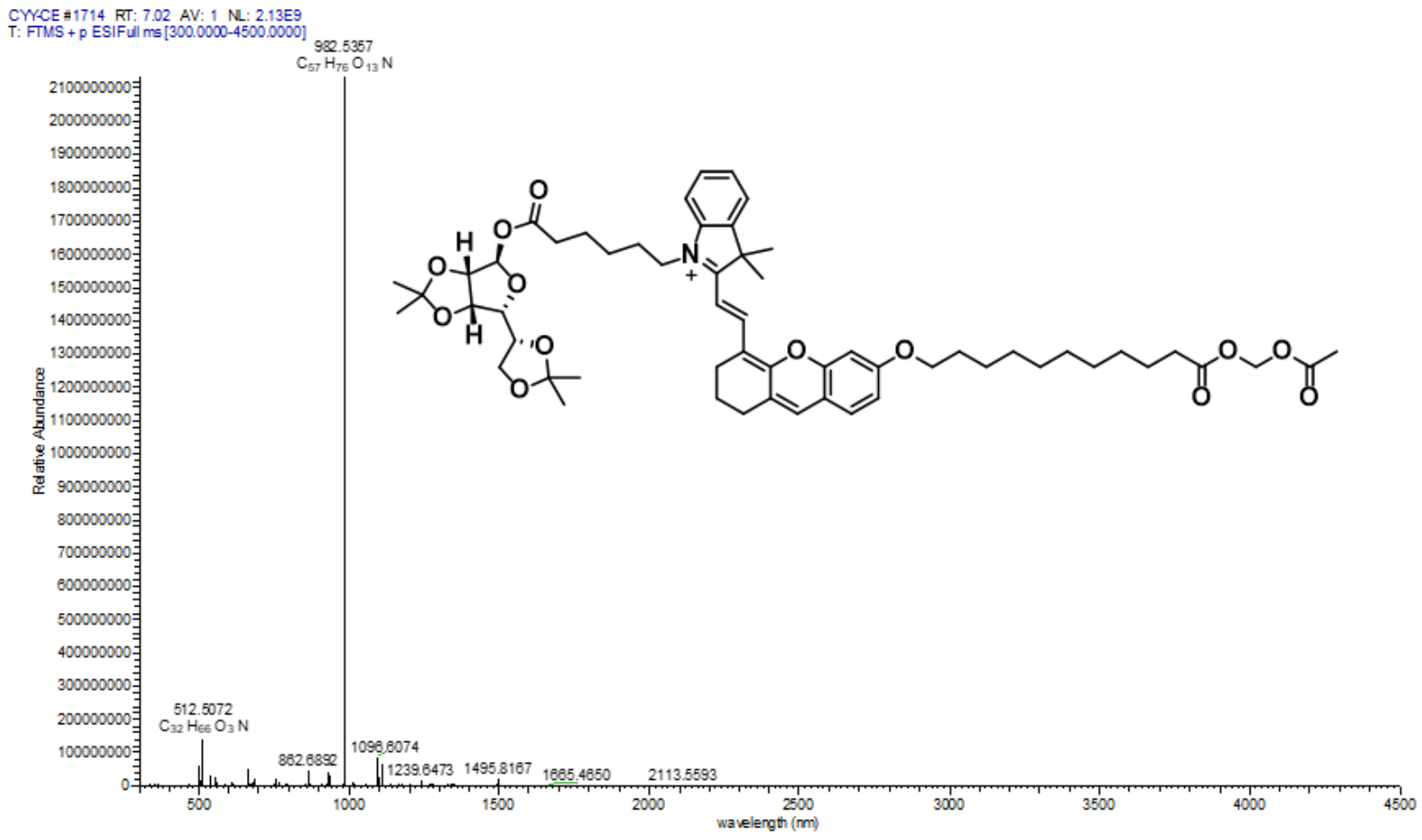

m/z	Theo. Mass	Delta (ppm)	RDB equiv.	Composition
982.5357	982.5311	4.67	20.5	C57 H76 O13 N

Figure S28. LC-MS results of compound 1-7.

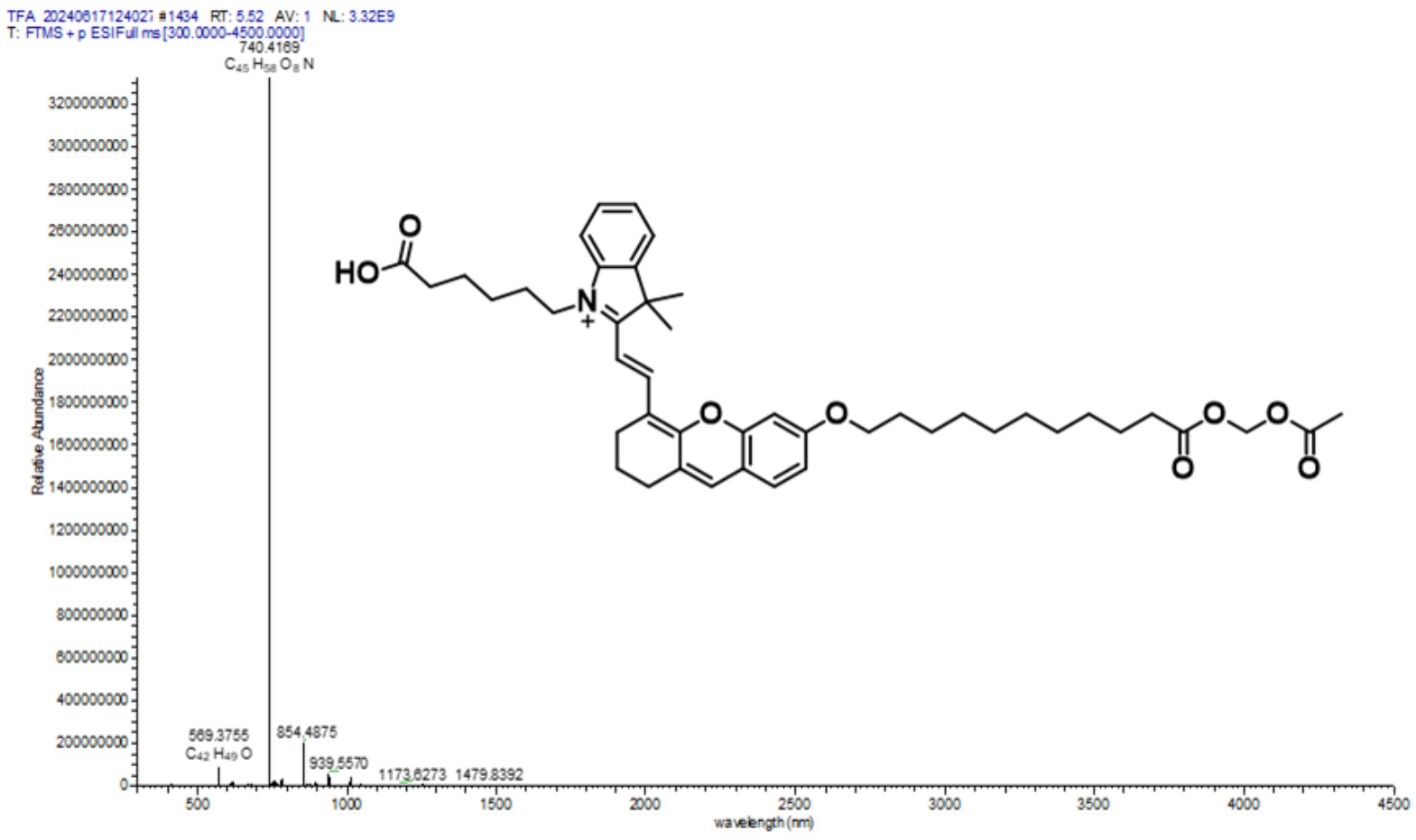

m/z	Theo. Mass	Delta (ppm)	RDB equiv.	Composition
740.4169	740.4157	1.59	17.5	C45 H58 O8 N

Figure S29. LC-MS results of compound 1-8.

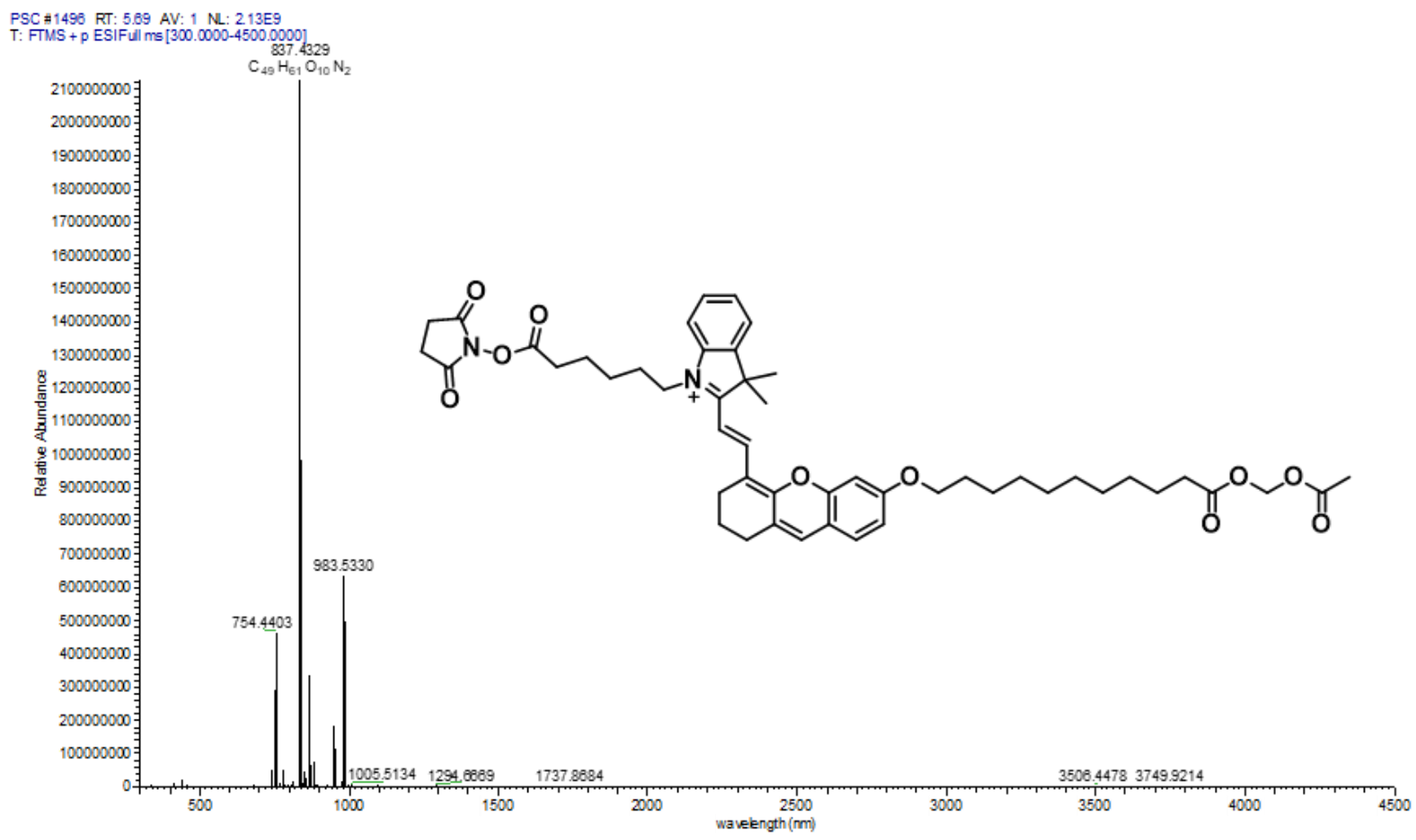

m/z	Theo. Mass	Delta (ppm)	RDB equiv.	Composition
837.4329	837.4321	0.94	20.5	C49 H61 O10 N2

Figure S30. LC-MS results of compound 1-9.

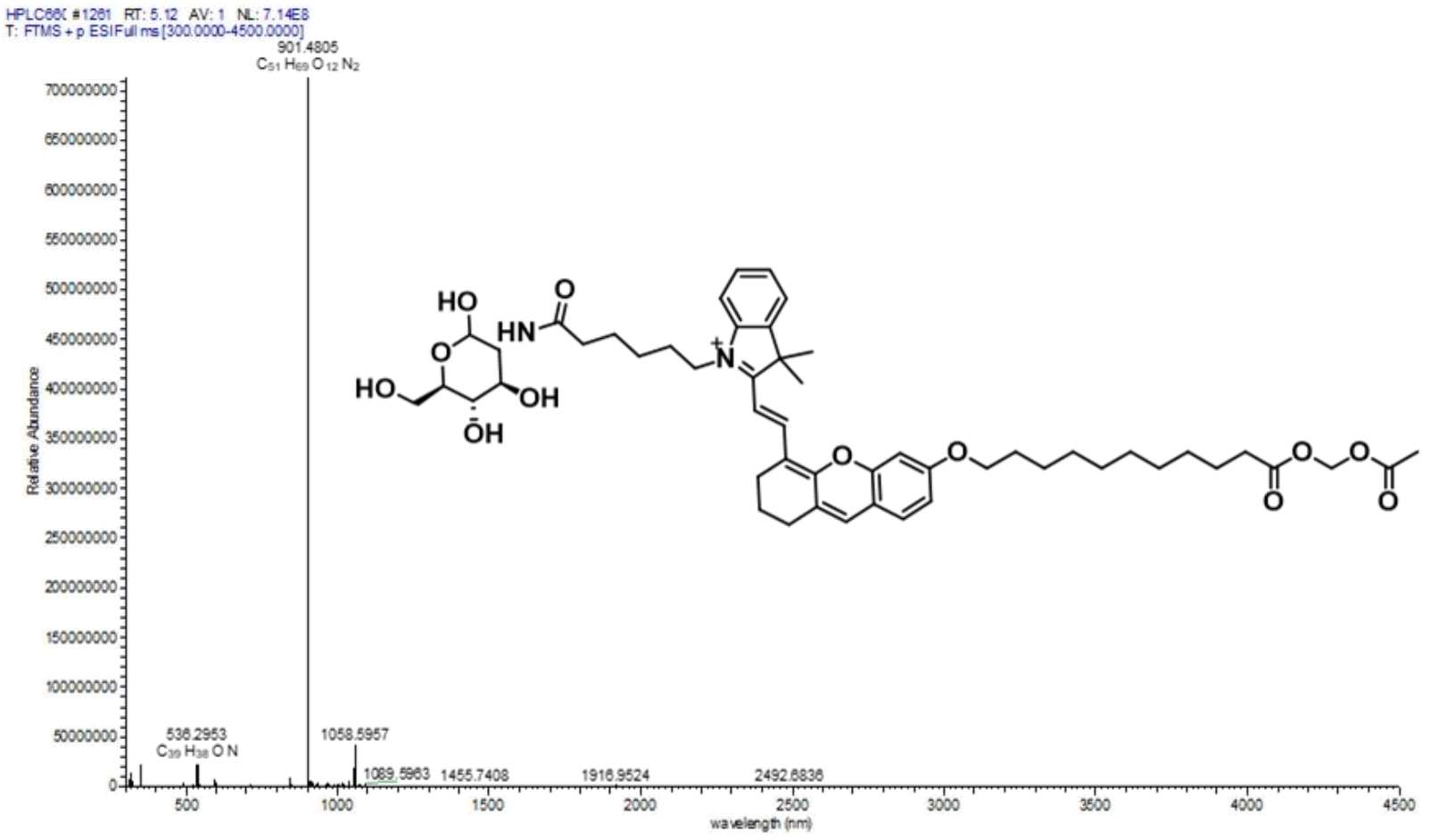

m/z	Theo. Mass	Delta (ppm)	RDB equiv.	Composition
901.4805	901.4845	-4.47	18.5	C51 H69 O12 N2

Figure S31. LC-MS results of compound **Dman-C11**.

Reference

[1] Y. J. Liu, X. H. Zheng, J. L. Zhou, Z. G. Xie, “Merocyanine-paclitaxel conjugates for photothermal induced chemotherapy,” *J. Mater. Chem. B* **9**, (2021): 2334–2340.
